# Streamlined ex vivo and in vivo genome editing in mouse embryos using recombinant adeno-associated viruses

**DOI:** 10.1038/s41467-017-02706-7

**Published:** 2018-01-29

**Authors:** Yeonsoo Yoon, Dan Wang, Phillip W. L. Tai, Joy Riley, Guangping Gao, Jaime A. Rivera-Pérez

**Affiliations:** 1 Department of Pediatrics, Division of Genes and Development, University of Massachusetts Medical School, 55 Lake Avenue North, Worcester, MA 01655 USA; 2 Department of Microbiology and Physiological Systems, University of Massachusetts Medical School, 55 Lake Avenue North, Worcester, MA 01655 USA; 3 Horae Gene Therapy Center, University of Massachusetts Medical School, 55 Lake Avenue North, Worcester, MA 01655 USA

## Abstract

Recent advances using CRISPR-Cas9 approaches have dramatically enhanced the ease for genetic manipulation in rodents. Notwithstanding, the methods to deliver nucleic acids into pre-implantation embryos have hardly changed since the original description of mouse transgenesis more than 30 years ago. Here we report a novel strategy to generate genetically modified mice by transduction of CRISPR-Cas9 components into pre-implantation mouse embryos via recombinant adeno-associated viruses (rAAVs). Using this approach, we efficiently generated a variety of targeted mutations in explanted embryos, including indel events produced by non-homologous end joining and tailored mutations using homology-directed repair. We also achieved gene modification in vivo by direct delivery of rAAV particles into the oviduct of pregnant females. Our approach greatly simplifies the generation of genetically modified mice and, more importantly, opens the door for streamlined gene editing in other mammalian species.

## Abstract (web summary)

CRISPR-Cas9 has been widely adopted for genetically manipulating rodents for scientific research. Here the authors transduce mouse embryos with CRISPR-Cas9 components using rAAVs in explant culture or in vivo to produce gene-edited animals.

## Introduction

The advent of clustered regularly interspaced short palindromic repeats-Cas9 (CRISPR-Cas9) gene editing technology has revolutionized gene targeting approaches and greatly facilitates the generation of genetically modified mice^1–3^. Despite the impressive advances in genome editing technology, methods to deliver nucleic acids into pre-implantation embryos has undergone minimal change. The conventional method, developed more than 30 years ago, relies on microinjection of zygotes to introduce RNA or DNA constructs^4,5^. This technique, however, requires sophisticated micromanipulation equipment that is operated by specially trained personnel^6,7^. A recently developed method relies on electroporation to deliver CRISPR-Cas9 components into zygotes^8–14^. This method has significantly improved the ability to generate gene-edited mice, yet it requires specialized equipment to electroporate embryos or the oviduct of pregnant females. An alternative approach is to use lentiviral vectors^15^. Nonetheless, lentivirus-based vectors have been shown to non-specifically integrate into the host genome, limiting their utility as an effective tool for generating transgenic mice. Furthermore, lentiviruses are unable to transduce pre-implantation embryos unless they are injected into the perivitelline space or the zona pellucida is removed prior to infection^15,16^.

Previous reports suggest that the zona pellucida is permeable to several wild-type viruses including adeno-associated viruses^17,18^. Here we explore the possibility of using recombinant adeno-associated viruses (rAAVs) as vehicles for transducing intact mouse zygotes to drive embryonic gene editing. AAV-based vectors offer several advantages: they can lead to high levels of transgene expression when delivered into host tissues, their genomes are predominantly episomal once unpackaged in the host cell, and they are rapidly diluted following cell division. In addition, the relatively low genotoxicity profile of rAAVs has also been extensively exploited in human gene therapy applications^19–21^. Our experiments indicate that multiple rAAV serotypes can permeate the zona pellucida and transduce intact mouse embryos at several pre-implantation stages independently of the mouse strain used. We also show that genetically modified mice can be generated after rAAV-driven transduction of zygotes with CRISPR-Cas9 expression cassettes in explant culture and subsequent transfer into pseudopregnant females. Moreover, we provide proof-of-principle evidence that in vivo CRISPR-Cas9 gene editing can be achieved by the simple injection of rAAVs into the oviduct of pregnant females. Our technology offers a viable alternative to current techniques that is not dependent on microinjection or electroporation of pre-implantation embryos. Furthermore, our in vivo approach obviates the need to isolate zygotes and the necessity to transfer treated embryos into pseudopregnant females, greatly simplifying the generation of genetically modified mice.

## Results

### Multiple rAAV serotypes transduce pre-implantation embryos

To determine if rAAV vectors can permeate the zona pellucida, we evaluated the ability of 14 rAAV serotypes to transduce explanted pre-implantation embryos. Intact eight-cell morulae were treated with a panel of rAAV serotypes packaged with an identical enhanced green fluorescent protein (EGFP) transgene (rAAV.CB6-*EGFP*) at a dose of ~9.0 × 10^9^ genome copies (GCs) and evaluated after 1 day in culture. EGFP fluorescence analysis showed that all the serotypes tested were capable of transducing intact morulae (Table 1, Supplementary Fig. 1). Serotype 6 was one of the most effective AAVs, showing high levels of fluorescence and embryo survival rate, and was therefore chosen for subsequent experiments. We successfully utilized rAAV6.CB6-*EGFP* to transduce zygotes from two inbred strains (C57BL/6NJ and FVB/N) and one outbred strain (CD-1) with 100% efficiency (Table 2). These results suggest that rAAVs can transduce intact mouse embryos at multiple pre-implantation stages, irrespective of mouse strain.

**Table 1:** Analysis of multiple rAAV serotypes for transduction of morulae ex vivo

Serotype^a^	Number of treated embryos	Number of surviving^b^ embryos (%)	Number of EGFP-positive embryos (%)	EGFP intensity^c^
rAAV1	8	7 (87)	3 (43)	+
rAAV2	9	9 (100)	9 (100)	+
rAAV3b	9	4 (44)	1 (25)	++
rAAV4	10	9 (90)	2 (22)	++
rAAV5	9	6 (67)	2 (33)	+
rAAV6	13	13 (100)	13 (100)	++++
rAAV6.2	11	7 (64)	7 (100)	+++
rAAV7	16	14 (87)	14 (100)	++++
rAAV8	17	14 (82)	8 (57)	++
rAAV9	12	9 (75)	2 (22)	++
rAAVrh.39	12	11 (92)	7 (63)	++++
rAAVrh.43	15	13 (87)	13 (100)	++++
rAAVrh.8	10	7 (70)	4 (57)	++
rAAVrh.10	13	11 (85)	9 (81)	+
no rAAV	81	74 (91)	0 (0)	n/a

^a^ Each rAAV serotype carries the same *EGFP*-expressing cassette

^b^ Embryos that developed to compacted morula or blastocyst stage after 1 day in culture

^c^ EGFP intensity was determined relative to non-treated control embryos and evaluated by two observers

**Table 2:** Transduction efficiency of zygotes from different strains of mice with rAAV6-*EGFP*

Genetic background	Treatment	Number of treated zygotes	Number of surviving^b^ embryos (%)	Number of EGFP-positive embryos (%)
C57BL/6J	rAAV6-*EGFP*^*a*^	30	30 (100)	30 (100)
	no rAAV	8	5 (63)	0 (0)
FVB/N	rAAV6-*EGFP*	24	16 (67)	16 (100)
	no rAAV	12	11 (92)	0 (0)
CD-1	rAAV6-*EGFP*	9	9 (100)	9 (100)
	no rAAV	8	7 (88)	0 (0)

^a^ Experimental embryos were exposed to viral particles for 5–6 h

^b^ Embryos that developed to compacted morula or blastocyst stage after 3 days in culture

### rAAV vectors can induce Cre-LoxP recombination in embryos

**Fig. 1:**
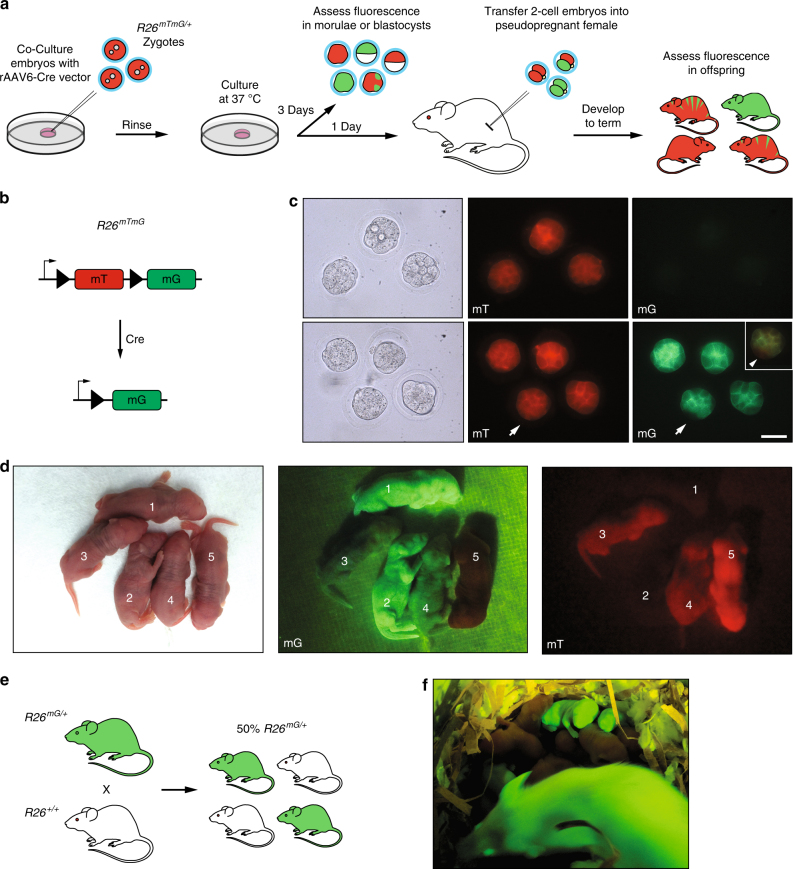
Recombinant AAV vectors can induce Cre-LoxP recombination. **a** Schematic representation of the strategy to induce Cre-LoxP recombination using rAAVs. *R26*^*mTmG*^ heterozygous zygotes derived from breeding *R26*^*mTmG*^ homozygous and wild-type mice were placed in a drop of KSOM media containing rAAV particles, rinsed, cultured in KSOM, and analyzed for fluorescence after 3 days in culture or were transferred into pseudopregnant females after 1 day in culture and allowed to develop to term. **b** Schematic representation of the *R26*^*mTmG*^ fluorescence reporter. *R26*^*mTmG*^ embryos express the membrane-targeted *tdTomato* gene (*mT*) and fluoresce red. Cre-mediated recombination drives expression of membrane-targeted *EGFP* (*mG*), making recombined cells fluoresce green. **c** Fluorescence analysis of compacted morulae transduced with rAAV6-*Cre*. Maternal mT protein is present in both non-treated (top row) and treated (bottom row) embryos, making them fluoresce red. Transduction with rAAV6-*Cre* leads to green fluorescent embryos (bottom row), indicative of Cre-mediated recombination. Inset is a merged image of the embryo marked by arrows to highlight mosaicism evident by the absence of green fluorescence in some cells. Scale bar equals 50 μm. **d** Fluorescence analysis of pups derived from zygotes transduced with rAAV6-*Cre* in culture and transferred into pseudopregnant females. Two pups show complete Cre-lox recombination (1 and 2), two are mosaic (3 and 4), and one (5) did not undergo recombination. **e** Schematic representation of the strategy to test for germline transmission of the recombined *R26*^*mG*^ allele obtained after rAAV6-*Cre* treatment of *R26*^*mTmG/+*^ zygotes in culture. **f** *R26*^*mG/+*^ mother and her offspring derived from a cross to a wild-type male; two *R26*^*mG/+*^ pups are visible

To demonstrate the feasibility of rAAVs to mediate germline transgenesis, we transduced *R26*^*mTmG*^ heterozygous zygotes with rAAV6.CB6-*Cre* (rAAV6-*Cre*) (Fig. 1a). The *R26*^*mTmG*^ reporter drives ubiquitous expression of membrane-bound tdTomato fluorescent protein. After Cre recombination, the tdTomato gene is excised and the EGFP gene is expressed^22^ (Fig. 1b). After treatment with rAAV6-*Cre* and 3 days in culture, the majority of *R26*^*mTmG*^ zygotes (32/38, 84%) underwent Cre recombination (Fig. 1c, Table 3). In addition, transfer of treated embryos into pseudopregnant females resulted in 37 out of 38 pups (97%) showing green fluorescence (Fig. 1d, Table 3, Supplementary Fig. 2). We tested two of these mice, one male and one female, for their ability to transmit the recombined transgene through the germline (Fig. 1e). Both founder mice produced multiple green fluorescent pups after mating to wild-type CD-1 mice, at a frequency close to the expected 50% Mendelian ratio (7/15 and 6/14, respectively) (Fig. 1f). These results show that rAAV6 particles can efficiently deliver Cre recombinase to zygotes to induce genetic recombination that is germline transmissible.

**Table 3:** Ex vivo Cre recombination after transduction of *R26*^*mTmG*^ zygotes with rAAV6-*Cre*

Number of treated zygotes	Time of analysis	Number of surviving embryos^a^ or pups (%)	Number of EGFP-positive embryos or pups (%)	Number of mosaic embryos or pups (%)
40	E3.5	38 (95)	32 (84)	5 (16)
74	P2^b^	38 (51)	37 (97)	15 (37)

^a^ Embryos that developed to compacted morula or blastocysts after 3 days in culture

^b^ Embryos were cultured overnight, transferred into pseudopregnant females and analyzed at postnatal day 2

### rAAV vectors induce CRISPR-Cas9 genome editing in embryos

To assess the ability of rAAV vectors to deliver *Cas9* and single-guide RNA (sgRNA) transgenes into intact zygotes and drive gene editing, we chose to target *Tyrosinase* (*Tyr*), a gene essential for the synthesis of melanin. This strategy provides a convenient way to visually screen for gene knockouts, since the bi-allelic inactivation of *Tyr* by insertions and/or deletions (indels) in embryos leads to albinism^23–25^. To express Cas9, we used rAAV6.U1a-Sp*Cas9* (rAAV6-*Cas9*), a vector containing the *Streptococcus pyogenes Cas9* gene driven by the mouse U1a snRNA promoter. A second vector, rAAV6.U6-sg*Tyr*-CB6-*EGFP* (rAAV6-sg*Tyr*), was used to drive the expression of a sgRNA under the control of the U6 promoter (Supplementary Fig. 3a). The rAAV6-sg*Tyr* vector also contains a cassette expressing *EGFP* under the control of the CB6 promoter to monitor transduction efficiency. We screened five sgRNAs targeting *Tyr* exon 1 and chose the most effective one, which targets a site located between the *Tyr*^*c-2J*^ mutation^23^ and the classic *Tyr*^*c*^ albino point mutation^24^, for subsequent experiments (Supplementary Fig. 3b–e).

**Fig. 2:**
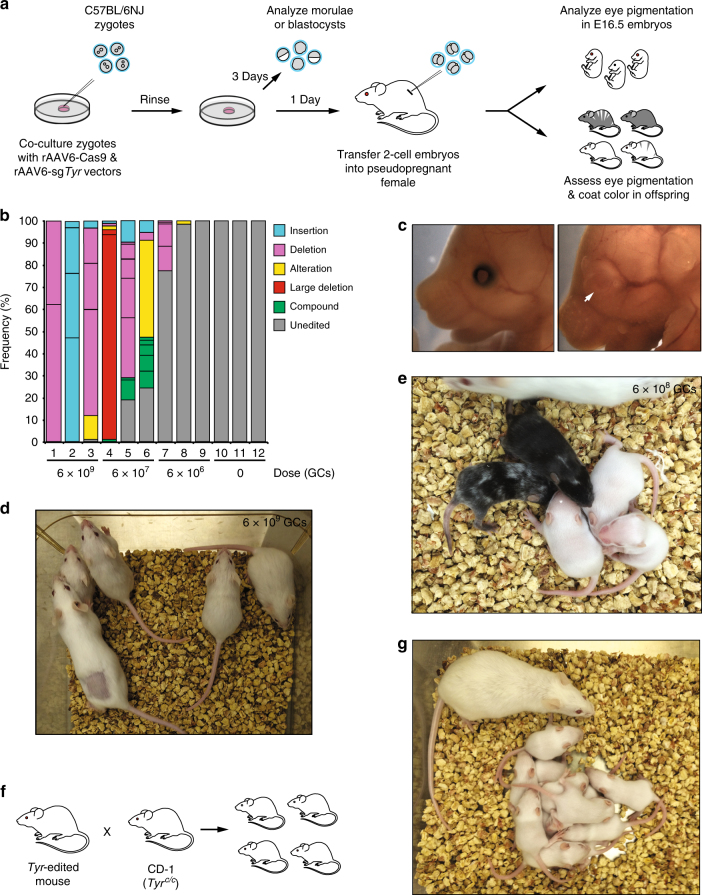
Gene editing in intact pre-implantation embryos transduced with rAAV vectors. **a** Schematic representation of the strategy to transduce C57BL/6NJ zygotes with rAAV vectors designed to target the *Tyrosinase* (*Tyr*) locus. Zygotes were placed in KSOM containing rAAV6-*Cas9* and rAAV6-sg*Tyr* vectors, rinsed, cultured at 37 °C for 3 days, and analyzed for *Tyr* gene editing at compacted morula or blastocyst stages. Alternatively, they were cultured overnight and transferred at the 2-cell stage into the oviducts of E0.5 pseudopregnant females. Transferred embryos were assessed for eye pigmentation at E16.5, or allowed to develop to birth and assessed for eye and coat color pigmentation. **b** Stacked histogram showing the percentage distribution of indel-type frequencies among four rAAV-dosage groups . Alterations indicate base replacements; large deletions are defined as removal of >20 bases and compound mutations are combinations of insertions, deletions, and/or alterations. **c** Analysis of eye pigmentation in E16.5 embryos transduced with rAAV6-*Cas9* only (left panel) and both rAAV6*-Cas9* and rAAV6*-*sg*Tyr* (right panel). Arrow in the right panel indicates the location of the eye in a transduced albino embryo. **d** Albino litter generated after transduction of C57BL/6NJ zygotes with CRISPR-*Cas9* rAAV vectors at 6 × 10^9^ GC dose. Shaved area on female indicates site of embryo transfer surgery. **e** A representative litter obtained after transduction of C57BL/6NJ zygotes with CRISPR-Cas9 rAAV vectors at 6 × 10^8^ GC dose. Three out of five pups are albino and two are mosaic as revealed by the variegated coat color pattern. **f** Schematic representation of the strategy to test germline transmission of CRISPR-Cas9-induced alleles of *Tyr*. *Tyr*-edited albino mice were mated with albino CD-1 (*Tyr*^*c/c*^) animals and the offspring were assessed for the presence of albino coat color. **g** Litter derived from *Tyr*-edited albino male crossed with a CD-1 female. All pups are albino indicating germline transmission

C57BL/6NJ zygotes were incubated with a 1:1 mixture of rAAV6-*Cas9* and rAAV6-sg*Tyr* at three vector doses (6 × 10^9^, 6 × 10^7^, and 6 × 10^6^ GCs) and cultured for 3 days until they reached the compacted morula or blastocyst stages (Fig. 2a). The prevalence of *Tyr* indel mutations in E3.5 embryos was determined by T7EI nuclease analysis, TOPO sequencing, and single-molecule real-time (SMRT) sequencing^26^ (Fig. 2b, Supplementary Fig. 4a and 5). We found evidence of indels in all experimental groups that was dosage related (Fig. 2b). Analysis of three embryos treated with the highest dose (6 × 10^9^ GCs) revealed indels in all three embryos with a frequency of 100% or ≥99% indels within each embryo. At the lowest dose (6 × 10^6^ GCs), two of three embryos showed evidence of indels. However, the indel incidence in these embryos was only 23% and 1.7%, indicating incomplete gene modification. At the intermediate dose (6 × 10^7^ GCs), all three embryos contained indels; one with an indel frequency of 100%, while the other two had frequencies of 80.9% and 75.6%. Note that multiple indel events exist in individual embryos, and that the variety of indels appears to be more prominent in the intermediate dosage group (upwards of eight indel types within a single embryo) than in the highest or lowest dosage groups (Fig. 2b). These results suggest that higher rAAV doses lead to indel events at earlier stages of development, while lower doses lead to gene modification at later stages. In fact, the presence of eight different indels in two of the embryos from the intermediate dose group suggests that CRISPR-Cas9 activity was present at or beyond the four-cell stage.

To gauge the fidelity of rAAV delivery of CRISPR-Cas9 components, we also conducted experiments to generate indels using microinjection. C57BL/6NJ zygotes were injected with either a high-dose mixture (100 ng/μl *Try* sgRNA and 50 ng/μl Cas9 RNA) or a low-dose mixture (5 ng/μl *Try* sgRNA and 6.67 ng/μl Cas9 RNA) of CRISPR-Cas9 reagents (Supplementary Fig. 6). These concentrations were based on previous reports of gene editing at the *Tyrosinase* locus^12,25^. Non-injected embryos were used as negative controls. Embryos were cultured to compacted morulae or blastocyst stages, and three embryos from each group were subjected to whole-genome amplification and SMRT sequencing. All three embryos belonging to the high-dose group showed a variety of indels that ranged from 2 to 13 different indel events per embryo (Supplementary Fig. 6). At the lower concentration, only two of the embryos showed evidence of indels, exhibiting 6 and 7 different indels. No evidence of indels was found in non-injected control embryos. These experiments reveal a variety of mutations per embryo indicative of mosaicism and suggest that rAAV-delivery and microinjection lead to similar genome editing outcomes.

### Generation of gene-edited mice after rAAV exposure ex vivo

To determine the ability of rAAV*-CRISPR-Cas9*-treated embryos to develop to birth, transduced zygotes were cultured overnight and those that advanced to the two-cell stage were transferred into pseudopregnant recipients. Embryos at E16.5 and newborns were assessed for the absence of eye pigmentation (Fig. 2c); 1-week old pups or older were also evaluated for albino coat color. The frequency of indels (defined as the percentage of phenotypically albino and mosaic animals) was 100% in embryos and newborns for the 6 × 10^9^ GC dose group (Table 4). Correspondingly, all of the pups generated with this dose were albino, suggesting bi-allelic targeting of *Tyrosinase* (Fig. 2d, Table 4 and Supplementary Fig. 4b, c). Zygotes treated with 6 × 10^8^ GCs resulted in live births with 100% indel frequency, among which 80% were albino (Fig. 2e and Table 4). The editing frequency dropped to 25% for E16.5 embryos and 20% for newborns at 6 × 10^7^ GCs. No edited animals were detected from the 6 × 10^6^ GC treatment group (Table 4). These results are consistent with the sequencing data obtained in E3.5 embryos (Fig. 2b), supporting the notion that gene editing efficiency is rAAV dose dependent.

**Table 4:** Ex vivo gene editing after transduction of C57BL/6NJ zygotes with CRISPR-Cas9 rAAV vectors

rAAV dosage (GCs)	Number of zygotes transferred	Time of analysis	Number of embryos or pups recovered (%)	*Tyr*-edited embryos or pups (albino)^a^	*Tyr* editing frequency (%)
6 × 10^9^	17	E16.5	7 (41)	7 (6)	100
	28	P10	5 (18)	5 (5)	100
6 × 10^8^	17	E16.5	9 (53)	7 (7)	78^b^
	46	P10	10 (22)	10 (8)	100
6 × 10^7^	35	E16.5	16 (48)	4 (0)	25
	83	P10	25 (30)	5 (3)	20
6 × 10^6^	16	P10	6 (38)	0	0
0	55	P10	19 (35)	0	0

^a^ Gene editing evidence obtained by assessing eye pigmentation in embryos or coat color in pups and by genome analysis

^b^ Two pigmented embryos were not assessed for gene editing at the genomic level

To test for germline transmission of *Tyr* indel alleles, one albino male and three albino females derived from the 6 × 10^9^ GC dose group were mated to albino CD-1 mice (*Tyr*^*c/c*^) (Fig. 2f). In these crosses, we only obtained albino pups from all of the tested founders, indicating successful germline transmission (Fig. 2g). These results show that gene modification mediated by rAAV delivery of *Cas9* and sgRNA transgenes is highly efficient and can lead to germline transmission.

It has been previously shown that the rAAV genome can integrate into the host genome following in vivo delivery in adult mice^27,28^. To analyze animals for rAAV genome integration, we performed PCR analysis to detect *Cas9* and *EGFP* genes using tail snip DNA from pups generated from transduced zygotes. The majority of samples (44/45) were negative, suggesting that episomal rAAV genomes present in zygotes were diluted during development and were eventually lost. However, one pup (1/45) was positive for both the *Cas9* and *EGFP* genes, indicating rAAV genome integration (Supplementary Fig. 7a). We reasoned that such rAAV genome integration would most likely occur at the Cas9 on-target cleavage site, which was confirmed by further analysis with a targeted PCR approach (Supplementary Fig. 7b, c). We additionally assessed the genomes of whole E16.5 embryos for genome-wide rAAV integration using a strategy comprising linear amplification mediated-PCR (LAM-PCR) and SMRT sequencing. No integration events were detected in rAAV6-*Cas9* control embryos or in the rAAV6-*Cas9*+rAAV6-*sgTyr* experimental group (*n* = 3) (Supplementary Fig. 8). Taken together, these results suggest that rAAV genome integration is a rare event in founder animals and can be identified by PCR-based genotyping.

### Small-scale preparations of rAAVs lead to gene-edited mice

The rAAV transduction experiments shown above were conducted using a traditional large-scale rAAV vector production procedure that requires specialized equipment and expertise^29^. However, we also achieved high-indel frequencies using a simplified small-scale protocol^30^ that can be adapted to standard molecular biology laboratories. This simplified protocol requires only a fifth of the producer cells required for large-scale production and a fraction of the rAAV purification time. The single-day purification procedure utilizes iodixanol gradient centrifugation and affordable purification columns, whereas the large-scale purification involves multiple runs of CsCl sedimentation followed by dialysis, and takes ~5 days to complete (Supplementary Fig. 9a). Using this small-scale protocol, we were able to produce rAAV vector titers (4.6 × 10^11^ GC/ml and 1.6 × 10^12^ GC/ml for sgTyr and sgFah, respectively), sufficient to generate indels in the *Tyr* and the *Fumarylacetoacetate hydrolase* (*Fah*) gene loci with 100% frequency (Supplementary Fig. 9b–e). Thus, genome editing can be efficiently achieved using small-scale preparation of rAAV vectors, and can be applied to more than one genomic locus.

### rAAV vectors generate precise genetic modifications via HDR

**Fig. 3:**
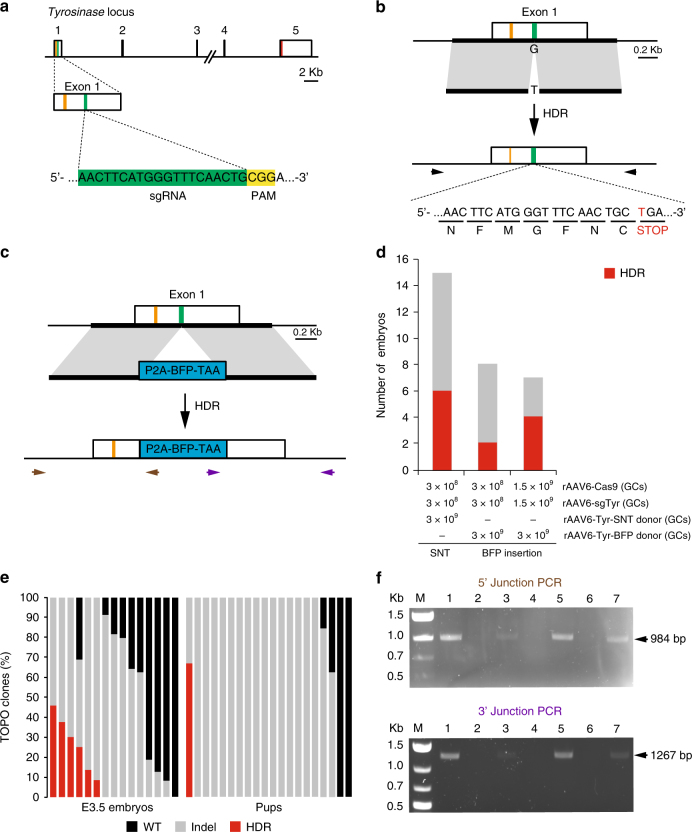
Recombinant AAV vectors can mediate homology-directed repair (HDR). **a** Schematic representation of the *Tyr* locus and location of sgRNA in exon 1. The orange and red lines mark the initiation and termination codons respectively. The green line indicates the location of the sgRNA used to target *Tyr*. **b** Strategy to introduce a premature stop codon in the *Tyr* locus using HDR. The 5′ and 3′ homology arms are marked by a thick line. A G to T nucleotide transversion in the PAM sequence converts a glycine codon (GGA) into a stop codon (TGA) disrupting translation of *Tyr*. Arrows indicate binding sites of the primers used in PCR-TOPO sequencing. **c** Strategy to insert the blue fluorescent protein (BFP) gene into the *Tyr* locus using HDR. Brown and purple arrows depict the binding sites of PCR primers used to confirm the insertion of BFP into *Tyr* locus. P2A, Porcine teschovirus-1 2A peptide; TAA, Stop codon. **d** Histogram showing the frequency of single-nucleotide transversion and BFP insertion by HDR using two different mixtures of rAAV vectors. **e** Analysis of single-nucleotide transversion in individual embryos or pups using PCR-TOPO sequencing. Each bar represents an individual sample. For pups, only DNA from tail snips and ear punches was analyzed. **f** Confirmation of BFP insertion using PCR. Four out of seven E3.5 embryos tested showed correct insertion of BFP into the *Tyr* locus. The top panel shows amplification of the 5′-junction of the targeted *Tyr* locus using a forward primer that binds to genomic DNA upstream of the homology region and a reverse primer that binds to the *BFP* gene as shown in (**c**). The bottom panel shows amplification of the 3′-junction of the *Tyr-*edited allele using a forward primer that binds to the *BFP* gene and a reverse primer that binds to genomic DNA downstream of the homology region

An important feature of genome editing is the ability to generate precise genetic changes. Therefore, we tested the capacity for rAAV vectors to deliver components for Cas9-mediated homology-directed repair (HDR). To achieve this goal, we designed two rAAV genomes as HDR donors for use in combination with the rAAV6-*Cas9* and rAAV6-sg*Tyr* vectors. The donor vectors carry a DNA construct that consists of ~800 bp homology arms on either side of the sg*Tyr* target site (Fig. 3a–c). A single-nucleotide transversion (SNT) strategy was used to generate a premature stop codon in *Tyr* for an albino phenotype (Fig. 3b). We also designed a donor vector to introduce a 771 bp blue fluorescent protein (BFP) cassette containing a porcine teschovirus-1 2A peptide and a stop codon (P2A-BFP-TAA) (Fig. 3c). We incubated zygotes with these three vectors and cultured them for 3 days until the compacted morulae or blastocyst stages for gene editing analysis. DNA obtained from SNT embryos was subjected to PCR, TOPO cloning, and Sanger sequencing to determine the frequency of the G to T transversion. We found that rAAV transduction resulted in 40% SNT-positive embryos (6/15) (Fig. 3d). Among the sequenced TOPO clones, the HDR frequency in individual SNT-positive embryos ranged from 8% to 45% (Fig. 3e). We also identified one live-born (1/20) that carried 68% SNT *Tyr* alleles (Fig. 3e). Mating this SNT-positive male with a CD-1 female resulted in 8 out of 15 (53%) pups carrying the SNT allele (Supplementary Fig. 10), indicating germline transmission.

The insertion of the BFP cassette was determined using PCR, and the BFP insertion was present in as high as 57% of embryos, depending on the vector dosage (Fig. 3d–f). We were also able to generate two pups that carried the BFP insertion (2/24) as determined by PCR analysis of tail snips and ear punches. One male was assessed for germline transmission by crossing to wild-type females. We found that 35% (9/26) of the F1 pups obtained from these crosses inherited the BFP insertion. These data demonstrate that infecting zygotes with rAAV vectors can induce HDR-mediated gene editing in embryos to generate genetically modified mice that can transmit the HDR allele to the next generation.

### rAAV-CRISPR-Cas9 vectors produce gene-edited mice in vivo

**Fig. 4:**
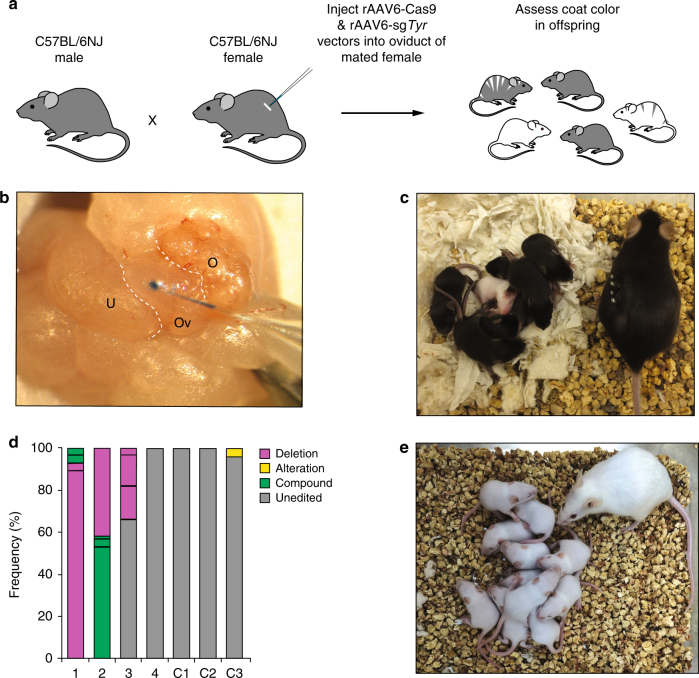
In vivo gene editing after oviduct injection with rAAV vectors. **a** Schematic representation of the strategy to induce in vivo gene editing of the *Tyr* locus. rAAV vectors carrying *SpCas9* and sg*Tyr* expression constructs were injected directly into the oviduct of plugged C57BL/6NJ females mated to C57BL/6NJ males. Coat color was assessed in the offspring. **b** Close-up view of the reproductive tract showing the process of in vivo delivery of rAAVs into the ampulla of the oviduct. The rAAV injection solution contains a blue tracer dye that is visible at the tip of the glass micropipette. A small pool of the injected solution is evident inside the ampulla. U uterus, Ov oviduct, O ovary. **c** Representative litter born after in vivo gene editing of *Tyr*. One out of eight pups born was albino. **d** Stacked histogram showing the percentage distribution of indel-type frequencies in two albino (1 and 2) and two black (3 and 4) pups by SMRT sequencing. Three C57BL/6NJ control samples (C1–C3) were included in the analysis. Alterations indicate base replacements; compound mutations are combinations of insertions, deletions, and/or alterations. The yellow area in sample C3 is likely the product of sequencing error. **e** Litter derived from *Tyr*-edited albino male crossed with a CD-1 female. All pups are albino indicating germline transmission

The ability of rAAV to mediate genetic modification of intact pre-implantation embryos ex vivo prompted the question of whether injection of viral particles into the oviduct of pregnant females could also result in gene modification of pre-implantation embryos in vivo. At E0.5, zygotes are located in the ampulla, a swollen region of the oviduct where fertilization occurs^6^. Therefore, we assessed the feasibility of in vivo embryonic gene modification by injecting rAAV6-*Cas9* and rAAV6-sg*Tyr* vectors directly into the ampulla of E0.5 C57BL/6NJ females mated with C57BL/6NJ males (Fig. 4a, b). We injected 1.5 to 3 μl of a solution containing a 1:1 mixture of the two vectors and Chicago sky blue dye (to visualize the site of injection) into the ampulla of a single oviduct in each female. We obtained 29 pups from 5 litters, and identified 3 pups with indels from 3 litters (Fig. 4c, d and Table 5). Gene modification was confirmed by SMRT sequencing (Fig. 4d). All three founder animals with indels (one male and two females) were tested for germline transmission by mating them with CD-1 albino mice and all three generated albino offspring indicating germline transmission (Fig. 4e). In summary, 3 out of 29 mice derived from in vivo embryonic transduction with rAAV vectors showed gene modification at the *Tyr* locus, a frequency of ~10% (Table 5). However, we note that because only one oviduct was injected per female, the gene editing frequency is likely an underrepresentation of the actual procedure efficiency.

**Table 5:** In vivo gene editing after injection of rAAVs into the oviduct of pregnant females

Vector injected^a^	Number of litters	Number of pups^b^	*Tyr*-edited pups (albino)^c^	*Tyr* editing frequency (%)^d^
rAAV6-SpCas9+rAAV6-sg*Tyr*	5	29	3 (2)	10

^a^ Only left oviduct was injected

^b^ Includes pups from injected and non-injected oviducts

^c^ Gene editing evidence determined by assessing coat color and sequence analysis

^d^ Approximate frequency; includes pups from treated and non-treated oviducts

These data suggest that rAAV particles can access pre-implantation embryos in the oviduct to deliver CRISPR-Cas9 components for genome modification in vivo.

## Discussion

There are several important ramifications for our study. First, to our knowledge, this is the first time that rAAV-based vectors have been used to transduce intact pre-implantation embryos in either ex vivo or in vivo in mice. Second, the ability to transduce embryos without removing the zona pellucida is a major advantage over using lentiviral vectors, which requires the removal of the zona pellucida, or microinjection into the perivitelline space^15,16^. Similarly, it is an advantage over electroporation techniques that require thinning of the zona pellucida with Tyrode’s solution to facilitate the penetration of the CRISPR-Cas9 reagents into the zygote^11–13^. Third, the use of rAAV vectors also enables the generation of genetically modified mice without relying on techniques that require sophisticated devices, such as pronuclear injection. Fourth, transduction with rAAV vectors allows for easy scale-up to modify large batches of embryos. Furthermore, we demonstrate that rAAVs can transduce pre-implantation embryos at the eight-cell stage. This is of particular relevance, since blastomeres at this stage are totipotent and offer an alternative to transducing zygotes^31,32^.

 Our technique offers an alternative approach that expands the toolbox for animal modeling. Using pronuclear injection, Yen et al.^25^ reported bi-allelic *Tyr* targeting in C57BL/6N zygotes (1 albino pup out of 33) using two sgRNAs while Chen et al.^12^ generated 88% albino mice by electroporating zygotes with SpCas9 protein and a single sgRNA targeting a *Tyr* site adjacent to ours. We also generated edits at the *Tyr* locus using pronuclear injection based on microinjection conditions reported in these two studies. Our results revealed high levels of indels in 5 out of 6 embryos and the presence of multiple indel events that ranged from 2 to 13 mutations per embryo. Our experiments using rAAVs led to 80% and 100% albino pups using doses of 6 × 10^8^ and 6 × 10^9^ GC rAAVs, respectively, suggesting comparable gene editing levels. However, it should be noted that, although the studies compared here share a common gene target in the same genetic strain of mice (the *Tyr* locus in C57BL/6 embryos), it is still difficult to make a fair comparison since multiple variables are at play. The experiments of Chen et al.^12^ provide a good example. These authors reported 88% bi-allelic targeting at the *Tyr* locus; however, much lower bi-allelic targeting was found at three other loci (*Cdh1*, 0%; *Kif1*, 14%; and *Cdk8*, 14%)^12^. These results suggest that factors other than the delivery method, such as differences in sgRNA choice, genomic environment, or inability to control the amount of CRISPR-Cas9 components that reach a zygote, can play roles in determining the final gene editing frequency in mouse embryos. Notably, we were able to achieve high targeting efficiency in *Tyr* as well as the *Fah* locus, supporting the robustness of our technique.

The occurrence of mosaicism in individual Cas9-edited founder animals appears to be a common phenomenon in genome editing strategies^12,25,33^, including ours. In pronuclear experiments, Yen et al.^25^ found that the majority of albino and mosaic founder animals harbored two or more *Tyr* mutant alleles^25^. Mosaicism was also found at the *Tyr* locus in electroporation studies^12^ and we also found a high level of mosaicism in our *Tyr* pronuclear injection experiments. SMRT sequencing analysis conducted in our rAAV experiments showed that levels of mosaicism appear to depend on the concentration of the rAAV dose used, with the lower dose (6 × 10^7^ GCs) resulting in a relatively high level of mosaicism. Interestingly, we observed that all three embryos of the high-dose group (6 × 10^9^ GCs), as well as two out of three embryos treated with the lower 6 × 10^7^ GC dose, harbored a dominant allele that accounted for about 50% of the total number of mutated alleles. Segregating such predominant mutant alleles in F1 animals is feasible following common breeding practices. Although mosaicism can be perceived as a negative outcome in gene editing experiments in mice, mosaic animals pose advantages under certain circumstances. For example, they can be useful in the establishment of mouse lines harboring lethal alleles^10^. This is an important point since it is estimated that 35.7% of knockout mice are lethal or sub-lethal before reaching weaning age^34^. Mosaics are also useful since they harbor a variety of mutations in the same locus, a valuable asset that can allow better dissection of gene function.

We detected one pup harboring rAAV integration at the Cas9 on-target cut site during analysis of 45 pups by performing PCR. Using a genome-wide analysis based on LAM-PCR, we did not detect rAAV genome integration in three E16.5 embryos tested. Therefore, rAAV integration appears to be a rare event that may be facilitated by Cas9-mediated DNA breaks. Intriguingly, this phenomenon could be exploited in homology-independent targeted integration events, as recently described^35^. Regardless, unwanted rAAV integration events at the target site can be circumvented by analyzing multiple independent mouse lines generated during the genome editing process. In addition, random rAAV integration events can be eliminated by consecutive breeding with wild-type animals.

Our results show that rAAVs can be used to introduce components for Cas9-mediated homology-directed repair. This is a major achievement that allows for the generation of specific genetic changes at precise genomic loci. Using traditional pronuclear injection techniques, Mizuno et al.^36^ generated a point mutation to mimic the *Tyr*^*c-2j*^ mutation using HDR via an oligo DNA donor; this led to 18% of pups carrying the HDR allele. Chen and co-workers also used oligo donor templates to generate point mutations at a site adjacent to our *Tyr* targeting site, with a frequency of 42%, or insertion of a 42 bp V5 tag at the *Sox2* locus with a frequency of 31%. In our study, introducing a point mutation in *Tyr* using a rAAV donor vector led to a frequency of 40% in E3.5 embryos. Using a different donor vector, we were able to introduce a 771 bp BFP cassette into the *Tyr* locus with a frequency in embryos as high as 57%. Surprisingly, a lower percentage of live-born animals carried such HDR events (1/20 and 2/24 for the point mutation and BFP insertion, respectively). One possible explanation is that these founder mice are mosaic and that the analysis of tail snips and ear punches may have led to an underestimation of HDR in the whole organism. This would not be the case in embryos where the whole animal was subjected to analysis. Another explanation is that HDR events present in progenitors of extra-embryonic tissues that are detected in the analysis of pre-implantation stage embryos will not be present in live borns. Nevertheless, we successfully generated F1 animals carrying the HDR alleles following a common breeding scheme, demonstrating that our approach is a viable alternative for generating HDR-mediated genetic modifications in mouse embryos for animal modeling.

We provide proof-of-concept demonstration that embryos can be genetically modified in vivo by direct delivery of rAAV particles into the oviduct. This is a tantalizing discovery that could further facilitate gene editing in mice and other mammalian species, since it will no longer require embryo isolation, microinjection, embryo culture, and transfer into pseudopregnant females. Oviduct injections are also simpler than performing oviduct or uterine transfers of embryos, a requirement for generating genetically modified mice using standard approaches. With the rapid development of modified CRISPR-Cas9 systems, it is possible to further enhance the efficiency and precision for our in vivo genome editing strategy. For example, a single vector platform expressing both a shorter version of Cas9 and target-specific sgRNA can be used to further simplify and improve our embryonic gene editing approach^1–3,37^. It may also be possible to combine our technology with multiple innovative approaches such as the use of mice constitutively expressing Cas9^38^ or base editing approaches to modify single bases in the genome^39^. Finally, one exciting possibility is the use of rAAVs to introduce large fragments of DNA into the genome by sequential homologous recombination  as recently described^40^. This is a clever achievement that significantly amplifies the potential of rAAV in genome editing studies in embryos.

## Methods

### Mouse strains and embryo collection

All animal experiments were conducted under the guidance of the institutional animal care and use committee of the University of Massachusetts Medical School. C57BL/6NJ (Stock No. 005304), FVB/NJ (Stock No. 001800), and *R26*^*mTmG*^ (*Gt(ROSA)26Sor* ^*tm4(ACTB-tdTomato,-EGFP)Luo*^, Stock No. 007676) mice were obtained from The Jackson Laboratory. CD-1 mice were obtained from Charles River Laboratories (Strain code 022). *R26*^*mTmG*^ mice were maintained in a CD-1 outbred genetic background. All animals were maintained in a 12 h light cycle. The middle of the light cycle of the day when a mating plug was observed was considered embryonic day 0.5 (E0.5) of gestation. Zygotes were collected at E0.5 by tearing the ampulla with forceps and incubation in M2 medium containing hyaluronidase to remove cumulus cells. Eight-cell morulae were collected by flushing the oviduct with M2 medium at E2.5.

### Recombinant AAV vectors

All the rAAV serotypes used in morulae experiments contained the same rAAV.CB6-*EGFP* construct. The EGFP expression vector consists of the CB6 promoter (cytomegalovirus enhancer fused to the chicken β-actin promoter) driving EGFP expression^41^. rAAV6.CB6-*Cre* carries the *Cre* recombinase gene driven by the CB6 promoter. In rAAV6.U1a-Sp*Cas9*, expression of the *S. pyogenes Cas9* (Sp*Cas9*) is driven by the ubiquitous U1a promoter^42^. scAAV6.U6-sgRNA.CB6-*EGFP* carries two expression cassettes, one expressing the sgRNA targeting the *Tyrosinase* (*Tyr*) gene or the *Fah* gene under the U6 promoter, and the other expressing *EGFP* under the CB6 promoter.

### Recombinant AAV vector production and purification

Recombinant AAV vectors were produced by calcium phosphate triple transfection of plasmids in HEK293 cells. For large-scale preparation, approximately 8.5 × 10^8^ cells were transfected. rAAV was purified by two rounds of CsCl sedimentation followed by dialysis^29^, which took a period of 7 days. For small-scale preparation, approximately 1.7 × 10^8^ cells were transfected. rAAVs were purified using iodixanol gradient centrifugation^30^. Briefly, HEK293 cells were detached by vigorously shaking the culture vessel. The cell suspension underwent three cycles of freezing (dry ice/ethanol bath) and thawing (37 °C water bath) for cell lysis and subsequent benzonase treatment. After centrifugation, the supernatant was transferred to an ultracentrifugation tube containing a discontinued gradient of 15, 25, 40, and 60% of iodixanol (Accurate Chemical, Cat. No. AN1114542). Gradient centrifugation was carried out at 504,000 × *g* for 70 min at 20 °C. The rAAV vectors at the 40–60% interface were collected and subjected to desalting using a Zeba column (Thermo Fisher Scientific, Cat. No. 89894) and concentrated using an Amicon Filter Unit (EMD Millipore, Cat. No. UFC910024). The entire procedure can be finished within 1 day. All rAAV vectors were titrated by droplet digital PCR (ddCPR, Bio-Rad) for genomes and silver staining of capsid proteins.

### Transduction of pre-implantation embryos in explant culture

Zygotes or 8-cell morulae were incubated in 10 or 15 μl drops of KSOM (Potassium-Supplemented Simplex Optimized Medium, Millipore, Cat. No. MR-020P-5F) containing the following rAAV vectors: scAAV.CB6-*EGFP* (9.0 × 10^9^ GCs); scAAV6.CB6-*EGFP* (2.25 × 10^9^ GCs); rAAV6.CB6-*Cre* (3.75 × 10^9^ GCs); rAAV6.U1a-*SpCas9* (3.0 × 10^9^ GCs, 1.5 × 10^9^ GCs, 3.0 × 10^8^ GCs, 3.0 × 10^7^ GCs, or 3.0 × 10^6^ GCs); scAAV6.U6-sg*Tyr*.CB6-*EGFP* (3.0 × 10^9^ GCs, 1.5 × 10^9^ GCs, 3.0 × 10^8^ GCs, 3.0 × 10^7^ GCs, or 3.0 × 10^6^ GCs); rAAV6.*Tyr*DonorWithSNT.CB6-mCherry (3.0 × 10^9^ GCs); rAAV6.TyrDonorWithP2A-*BFP*.CB6-mCherry (3.0 × 10^9^ GCs); scAAV6.U6-sg*Fah*Exon.CB6-EGFP (3.0 × 10^9^ GCs, 3.0 × 10^8^ GCs) for 5–6 h. Drops were placed in 35 mm plates under mineral oil (Sigma, M8410) at 37 °C in a tissue culture incubator containing 5% CO_2_ and 5% O_2_. After the incubation period, the embryos were rinsed once in M2 medium and transferred to fresh KSOM for subsequent culture. Zygotes were cultured for 3 days and morulae for 1 day to reach compacted morula or blastocyst stages. To develop transduced zygotes to term, embryos were cultured overnight and those that advanced to the two-cell stage were transferred into the oviduct of E0.5 pseudopregnant CD-1 females. Operated females were allowed to carry the embryos to term or were euthanized at E16.5 to obtain embryos for analysis.

### Transduction of zygotes in vivo using rAAVs

Recombinant AAVs were injected into the oviduct of females on the day when the mating plug was observed (E0.5). Only the oviduct of the left horn was injected. The untreated right horn served as a hedge for pregnancy loss in the case of embryo lethality on the treated side of the oviduct. The volume injected ranged from 1.5 to 3 μl and was injected using glass needles with tip diameter of 15–30 μm. The tracer dye Chicago sky blue (0.1%) (Sigma Cat. No. C8679) was used to track the site of injection. To generate indels using the CRISPR-Cas9 system, we injected E0.5 C57BL/6NJ females mated to males of the same strain. A 1:1 mixture of rAAV6.U1a*-SpCas9* and scAAV6.U6-sg*Tyr*.CB6*-EGFP* (4.0 × 10^9^ GCs/μl each) was injected into the ampulla of the left oviduct. The right oviduct was not injected. Operated females were allowed to carry the embryos to term.

### Generation of gene-edited embryos using pronuclear injection

Zygotes derived from crosses between C57BL/6J mice were collected at E0.5 as described above. . The male pronucleus was microinjected using a high (100 ng/μl *Tyr* sgRNA and 50 ng/μl Cas9 RNA) or a low (5 ng/μl *Tyr *sgRNA and 6.67 ng/μl Cas9 RNA) dose. Injected zygotes were cultured overnight in KSOM media for 3 days until they reached the compacted morula or blastocyst stage. Embryos were then processed for whole-genome amplification and SMRT sequencing as described below.

### Analysis of embryos or pups transduced with rAAV6.CB6-*Cre*

To determine Cre-mediated recombination in transduced *R26*^*mTmG/+*^ embryos, EGFP fluorescence was assessed in morulae or blastocysts. Fluorescence was assessed qualitatively relative to non-transduced controls using an inverted Leica microscope (DMI4000) equipped with epifluorescence. Pups were screened at postnatal day 1 or 2 (P1 or P2) for the presence of EGFP (mG) or tdTomato (mT) fluorescence using a dual fluorescent protein flashlight (Nightsea, Bedford, MA. USA).

### Fluorescence imaging of adult tissue cryosections

Mice were anesthetized by isoflurane, and transcardially perfused with ice-cold phosphate-buffered saline followed by 4% paraformaldehyde (PFA). Organs were dissected and post-fixed in 4% PFA overnight. Organs were then cryopreserved in 30% sucrose overnight, embedded in Tissue-Tek OCT compound (Sakura Finetek), and sectioned at a thickness of 8 µm in a cryostat. Tissue sections were mounted with vectashield mounting medium containing DAPI (Vector Labs, H1200), and imaged using an upright fluorescence microscope (Leica DM5500B).

### Analysis of embryos or pups transduced with rAAVs

To determine the genotype of edited *Tyr* alleles, individual compacted morulae, blastocysts, or E16.5 embryos were collected and subjected to SMRT sequencing analysis or T7EI nuclease assay (see below). The phenotype was assessed at E16.5 or after birth. The levels of eye pigmentation in E16.5 embryos were determined using a dissection microscope (Leica MZ16F) equipped with color camera (Leica DFC420). For P2 or later pups, eye pigmentation and coat color were visually assessed.

### SMRT sequencing and bioinformatics analysis

Harvested embryos were subjected to whole-genome amplification using the REPLI-g Single Cell Kit (Qiagen, Cat No. 150343). A portion of the *Tyr* gene was amplified using the KOD Hot Start DNA Polymerase (EMD Millipore, Cat. No. 71086) and purified using the QIAquick PCR purification kit (Qiagen, Cat No. 28106). Primer pairs used for PCR were uniquely indexed for each embryo at the 5′ ends with 16-nucleotide asymmetric barcodes (see Supplementary Figure 5a for complete primer set list). PCR products were pooled for SMRTbell template preparation and sequenced using a PacBio RS II sequencer following standard guidelines and procedures by the University of Massachusetts Medical School, Deep Sequencing Core. Raw reads were processed by SMRT Analysis software (v2.3.0) pipelines to produce reads-of-inserts (ROIs) representing multiplexed PCR amplicon sequences in fastq format. All downstream workflows were performed using the Galaxy web-based platform for genome data analysis^44–46^, unless specified. Reads were filtered by length and demultiplexed. Sequences were then aligned with BWA-MEM^47^ to a custom reference representing the unedited, wild-type *Tyr* amplicon sequence. Imperfect alignments (deletions, insertions, and mismatches) across the predicted edit site (−3nt of the PAM) were designated as indel events. To determine the distribution of indel types due to Cas9 editing, only full and intact reads that encompassed both asymmetric barcodes were considered for analysis. Fasta formatted reads were clustered with USEARCH v8.1 sequence analysis tools^48^. Specifically, identical sequences were tabulated with the -derep_fulllength command, followed by sequence clustering using operational taxonomic units (OTUs) with the -cluster_otus command with the following options: -fulldp, -otu_radius_pct 0.1, -minsize 5, -gapopen *I/1.0E, and -gapext *I/0.5E. Sequence clusters were manually curated to group and count indel types. Unique indel types were scored as a percentage of total reads.

### DNA preparation for T7EI assay

GreenGo cells^49^ were co-transfected with pAAV.U1a-*SpCas9* and pAAVsc.U6-sg*Tyr*.CB6-EGFP using Lipofectamine 3000 Transfection Reagent (Thermo Fisher Sci. Cat. No. L3000015). Three days later, total DNA was extracted using the QIAamp DNA Mini Kit (Qiagen, Cat. No. 51306). Embryos cultured up to compacted morula or blastocyst stages were harvested and subjected to whole-genome amplification using the REPLI-g Single Cell Kit (Qiagen, Cat. No. 150343). Whole E16.5 embryos were stored at −80 °C until being powdered in liquid nitrogen. DNA was then extracted from tissue powder using the Blood & Cell Culture DNA Maxi Kit (Qiagen, Cat. No. 13362).

### T7EI nuclease assay

A portion of the *Tyr* gene or *Fah* gene was amplified using the KOD Hot Start DNA Polymerase (EMD Millipore, Cat. No. 71086), purified using the QIAquick PCR purification kit (Qiagen, Cat. No. 28106), and subjected to T7EI nuclease assay according to the manufacturer’s instruction (NEB, Cat. No. M0302L). Digested products were resolved on a 2% agarose gel containing ethidium bromide and imaged. Primers used for PCR are listed in Table 6.

**Table 6:** List of primers used in PCR analysis

Target	Orientation	Sequence
*Tyr*	Forward	5′-TTGTTGGCAAAAGAATGCTG-3′
	Reverse	5′-GCTTCATGGGCAAAATCAAT-3′
*Tyr* G to T transversion	Forward	5′-TGAAGCAGTTCACCAAAATAAC-3′
	Reverse	5′-CTGTTTGAGAGTCAGCAACG-3′
BFP/*Tyr* 5′ junction	Forward	5′-TGAAGCAGTTCACCAAAATAAC-3′
	Reverse	5′-GCGAGCTGATTAAGGAGAAC-3′
BFP/*Tyr* 3′ junction	Forward	5′-GCTAAGAACCTCAAGATGCC-3′
	Reverse	5′-CGTTGCTGACTCTCAAACAG-3′
*Fah*	Forward	5′-ACCCCTGTGTGATAGACCAC-3′
	Reverse	5′-CATGGGCTGCTATTTGTGGC-3′
*SpCas9*	Forward	5′-CTGAGCAAGGACACCTACGA-3′
	Reverse	5′-CTCGGTGTTCACTCTCAGGA-3′
*EGFP*	Forward	5′-CTGAAGTTCATCTGCACCACC-3′
	Reverse	5′-ATGCCGTTCTTCTGCTTGTCG-3′
Integration of rAAV	Forward	5′-AGGAACCCCTAGTGATGGAGT-3′
	Reverse	5′-GCTTCATGGGCAAAATCAAT-3′

### TOPO sequencing

PCR products were purified using the QIAquick PCR Purification Kit (Qiagen, Cat. No. 28106). Purified PCR products were cloned into the pCR^TM^-Blunt II-TOPO vector using Zero Blunt TOPO PCR Cloning Kit (Thermo Fisher Sci. Cat. No. K280002), and used to transform DH5α *Escherichia coli* bacteria. Plasmid from individual colonies was extracted using the QIAcube automated sample preparation station (Qiagen), and subjected to Sanger sequencing.

### Analysis of rAAV vector genome integration by standard PCR

DNA of tail snips was used for PCR analysis. The same DNA samples were also used for PCR and TOPO cloning and Sanger sequencing. The primer sequences are shown in Table 6. PCR was carried out using the KOD Hot Start DNA Polymerase (EMD Millipore, Cat. No. 71086), and amplified for 35 cycles. PCR products were resolved on a 1% agarose gel containing ethidium bromide and imaged. A band of predicted size (~450 bp) was excised and purified using the QIAquick Gel Extraction Kit (Qiagen, Cat. No. 28706). Purified PCR product was cloned into the pCR^TM^-Blunt II-TOPO vector using Zero Blunt TOPO PCR Cloning Kit (Thermo Fisher Sci. Cat. No. K280002) and sequenced using the Sanger method.

### Genome-wide rAAV vector integration analysis

DNA libraries for integration profiling were generated by LAM-PCR and subjected to SMRT sequencing. The overall protocol design was modified from the high-throughput, genome-wide, translocation sequencing procedure^50^. Briefly, whole-genomic DNAs were extracted from snap-frozen and powdered tissues from experimental E16.5 embryos, and adult mouse liver treated with rAAV9*-SpCas9* and rAAV9.U6-sg*Aspa*.CB6*-EGFP* as a positive control sample. Genomic material (20 μg total input) was fragmented by Taq^α^I digestion (NEB, Cat. No. R0149M). Fragmented DNAs were subjected to phenol–chloroform extraction and ethanol precipitation to purify the fragmented material. Template DNAs were next subjected to 80 cycles of LAM-PCR with KOD Hot Start DNA Polymerase and a biotinylated primer with specificity to the rAAV-polyA sequence: 5′-/5Biosg/CTTGAGCATCTGACTTCTGGCTAATAAAGG-3′. Single-strand, biotinylated PCR products were next captured on magnetic beads, enriched, and ligated to a bridge adapter by on-bead ligation. Nested PCR (30 cycles) to generate SMRT sequencing libraries was next carried out using asymmetrically barcoded forward and reverse primer sets:

Forward: 5′-XXXXXXXXXXXXXXXXAGGAACCCCTAGTGATGGAGT-3′

Reverse: 5′-XXXXXXXXXXXXXXXXACTATAGGGCACGCGTGGT-3′.

Individual libraries were then subjected to 0.6× AMPurePB bead (Pacific Biosciences, Cat. No. 100-265-900) purification, pooled, and submitted for standard SMRT sequencing analysis as described above. The resulting ROIs were filtered by barcode demultiplexing and screened for the presence of a 10-nt feature that is unique to the rAAV-ITR (5′-TGGCCACTCC-3′). This filtering method ensures that non-specific amplification products are not falsely identified as integration events. The resulting positive reads were then mapped to the mm10 mouse genome using BWA-MEM^47^. Integration events were summarized using a custom R-script (ggbio)^51^ to display as a karyogram.

### Data availability

Sequencing data have been deposited in the NCBI sequence read archive (SRA) under accession code 127366 . All other data are available from the authors upon reasonable request.

## Electronic supplementary material

Supplementary Information

## References

[1] Hsu PD, Lander ES, Zhang F (2014). Development and applications of CRISPR-Cas9 for genome engineering. Cell.

[2] Bolukbasi MF, Gupta A, Wolfe SA (2016). Creating and evaluating accurate CRISPR-Cas9 scalpels for genomic surgery. Nat. Methods.

[3] Komor AC, Badran AH, Liu DR (2017). CRISPR-based technologies for the manipulation of eukaryotic genomes. Cell.

[4] Palmiter RD (1982). Dramatic growth of mice that develop from eggs microinjected with metallothionein-growth hormone fusion genes. Nature.

[5] Brinster RL, Chen HY, Warren R, Sarthy A, Palmiter RD (1982). Regulation of metallothionein--thymidine kinase fusion plasmids injected into mouse eggs. Nature.

[6] Nagy A (2003). *Manipulating the Mouse Embryo: A Laboratory Manual*.

[7] Wang H (2013). One-step generation of mice carrying mutations in multiple genes by CRISPR/Cas-mediated genome engineering. Cell.

[8] Hashimoto M, Takemoto T (2015). Electroporation enables the efficient mRNA delivery into the mouse zygotes and facilitates CRISPR/Cas9-based genome editing. Sci. Rep..

[9] Takahashi G (2015). GONAD: genome-editing via oviductal nucleic acids delivery system: a novel microinjection independent genome engineering method in mice. Sci. Rep..

[10] Hashimoto M, Yamashita Y, Takemoto T (2016). Electroporation of Cas9 protein/sgRNA into early pronuclear zygotes generates non-mosaic mutants in the mouse. Dev. Biol..

[11] Qin W (2015). Efficient CRISPR/Cas9-mediated genome editing in mice by zygote electroporation of nuclease. Genetics.

[12] Chen S, Lee B, Lee AY, Modzelewski AJ, He L (2016). Highly efficient mouse genome editing by CRISPR ribonucleoprotein electroporation of zygotes. J. Biol. Chem..

[13] Wang W (2016). Delivery of Cas9 protein into mouse zygotes through a series of electroporation dramatically increases the efficiency of model creation. J. Genet. Genom..

[14] Gurumurthy CB (2016). GONAD: a novel CRISPR/Cas9 genome editing method that does not require ex vivo handling of embryos. Curr. Protoc. Hum. Genet..

[15] Lois C, Hong EJ, Pease S, Brown EJ, Baltimore D (2002). Germline transmission and tissue-specific expression of transgenes delivered by lentiviral vectors. Science.

[16] Pfeifer A, Ikawa M, Dayn Y, Verma IM (2002). Transgenesis by lentiviral vectors: lack of gene silencing in mammalian embryonic stem cells and preimplantation embryos. Proc. Natl. Acad. Sci. USA.

[17] Bowen RA (1979). Viral infections of mammalian preimplantation embryos. Theriogenology.

[18] Botquin V, Cid-Arregui A, Schlehofer JR (1994). Adeno-associated virus type 2 interferes with early development of mouse embryos. J. Gen. Virol..

[19] Vasileva A, Jessberger R (2005). Precise hit: adeno-associated virus in gene targeting. Nat. Rev. Microbiol..

[20] Asokan A, Schaffer DV, Samulski RJ (2012). The AAV vector toolkit: poised at the clinical crossroads. Mol. Ther..

[21] Samulski RJ, Muzyczka N (2014). AAV-mediated gene therapy for research and therapeutic purposes. Annu Rev. Virol..

[22] Muzumdar MD, Tasic B, Miyamichi K, Li L, Luo L (2007). A global double-fluorescent Cre reporter mouse. Genesis.

[23] Le Fur N, Kelsall SR, Mintz B (1996). Base substitution at different alternative splice donor sites of the tyrosinase gene in murine albinism. Genomics.

[24] Yokoyama T (1990). Conserved cysteine to serine mutation in tyrosinase is responsible for the classical albino mutation in laboratory mice. Nucleic Acids Res..

[25] Yen ST (2014). Somatic mosaicism and allele complexity induced by CRISPR/Cas9 RNA injections in mouse zygotes. Dev. Biol..

[26] Eid J (2009). Real-time DNA sequencing from single polymerase molecules. Science.

[27] Zhong L (2013). Recombinant adeno-associated virus integration sites in murine liver after ornithine transcarbamylase gene correction. Hum. Gene. Ther..

[28] Donsante A (2007). AAV vector integration sites in mouse hepatocellular carcinoma. Science.

[29] Gao, G. P. & Sena-Esteves, M. in *Molecular Cloning: A Laboratory Manual* (eds M. R, Green. & J, Sambrook) 1209–1313 (Cold Spring Harbor Laboratory Press, New York, 2012).

[30] Burger C, Nash KR (2016). Small-scale recombinant adeno-associated virus purification. Methods Mol. Biol..

[31] Balakier H, Pedersen RA (1982). Allocation of cells to inner cell mass and trophectoderm lineages in preimplantation mouse embryos. Dev. Biol..

[32] Kelly SJ (1977). Studies of the developmental potential of 4- and 8-cell stage mouse blastomeres. J. Exp. Zool..

[33] Yang H, Wang H, Jaenisch R (2014). Generating genetically modified mice using CRISPR/Cas-mediated genome engineering. Nat. Protoc..

[34] Dickinson ME (2016). High-throughput discovery of novel developmental phenotypes. Nature.

[35] Suzuki K (2016). In vivo genome editing via CRISPR/Cas9 mediated homology-independent targeted integration. Nature.

[36] Mizuno S (2014). Simple generation of albino C57BL/6J mice with G291T mutation in the tyrosinase gene by the CRISPR/Cas9 system. Mamm. Genome.

[37] Mitsunobu H, Teramoto J, Nishida K, Kondo A (2017). Beyond Native Cas9: manipulating genomic information and function. Trends Biotechnol..

[38] Platt RJ (2014). CRISPR-Cas9 knockin mice for genome editing and cancer modeling. Cell.

[39] Komor AC, Kim YB, Packer MS, Zuris JA, Liu DR (2016). Programmable editing of a target base in genomic DNA without double-stranded DNA cleavage. Nature.

[40] Bak RO, Porteus MH (2017). CRISPR-mediated integration of large gene cassettes using AAV donor vectors. Cell Rep..

[41] Wang H (2014). Widespread spinal cord transduction by intrathecal injection of rAAV delivers efficacious RNAi therapy for amyotrophic lateral sclerosis. Hum. Mol. Genet..

[42] Bartlett JS (1996). Efficient expression of protein coding genes from the murine U1 small nuclear RNA promoters. Proc. Natl. Acad. Sci. USA.

[43] Behringer R, Gertsenstein M, Nagy KV, Nagy A (2014). *Manipulating the Mouse Embryo: A Laboratory Manual*.

[44] Blankenberg D (2010). Galaxy: a web-based genome analysis tool for experimentalists. Curr. Protoc. Mol. Biol..

[45] Giardine B (2005). Galaxy: a platform for interactive large-scale genome analysis. Genome Res..

[46] Goecks J, Nekrutenko A, Taylor J, Galaxy T (2010). Galaxy: a comprehensive approach for supporting accessible, reproducible, and transparent computational research in the life sciences. Genome Biol..

[47] Li H, Durbin R (2009). Fast and accurate short read alignment with Burrows-Wheeler transform. Bioinformatics.

[48] Edgar RC (2010). Search and clustering orders of magnitude faster than BLAST. Bioinformatics.

[49] Sanchez-Rivera FJ (2014). Rapid modelling of cooperating genetic events in cancer through somatic genome editing. Nature.

[50] Frock RL (2015). Genome-wide detection of DNA double-stranded breaks induced by engineered nucleases. Nat. Biotechnol..

[51] Yin T, Cook D, Lawrence M (2012). ggbio: an R package for extending the grammar of graphics for genomic data. Genome Biol..

