# Supplementary material for: Streamlined ex vivo and in vivo genome editing in mouse embryos using recombinant adeno-associated viruses
Source: Nat Commun. 2018 Jan 29;9:412. doi: 10.1038/s41467-017-02706-7 (PMC5788975; doi:10.1038/s41467-017-02706-7)
Supplement: Supplementary file 1 — Supplementary Information [file 41467_2017_2706_MOESM1_ESM.pdf]

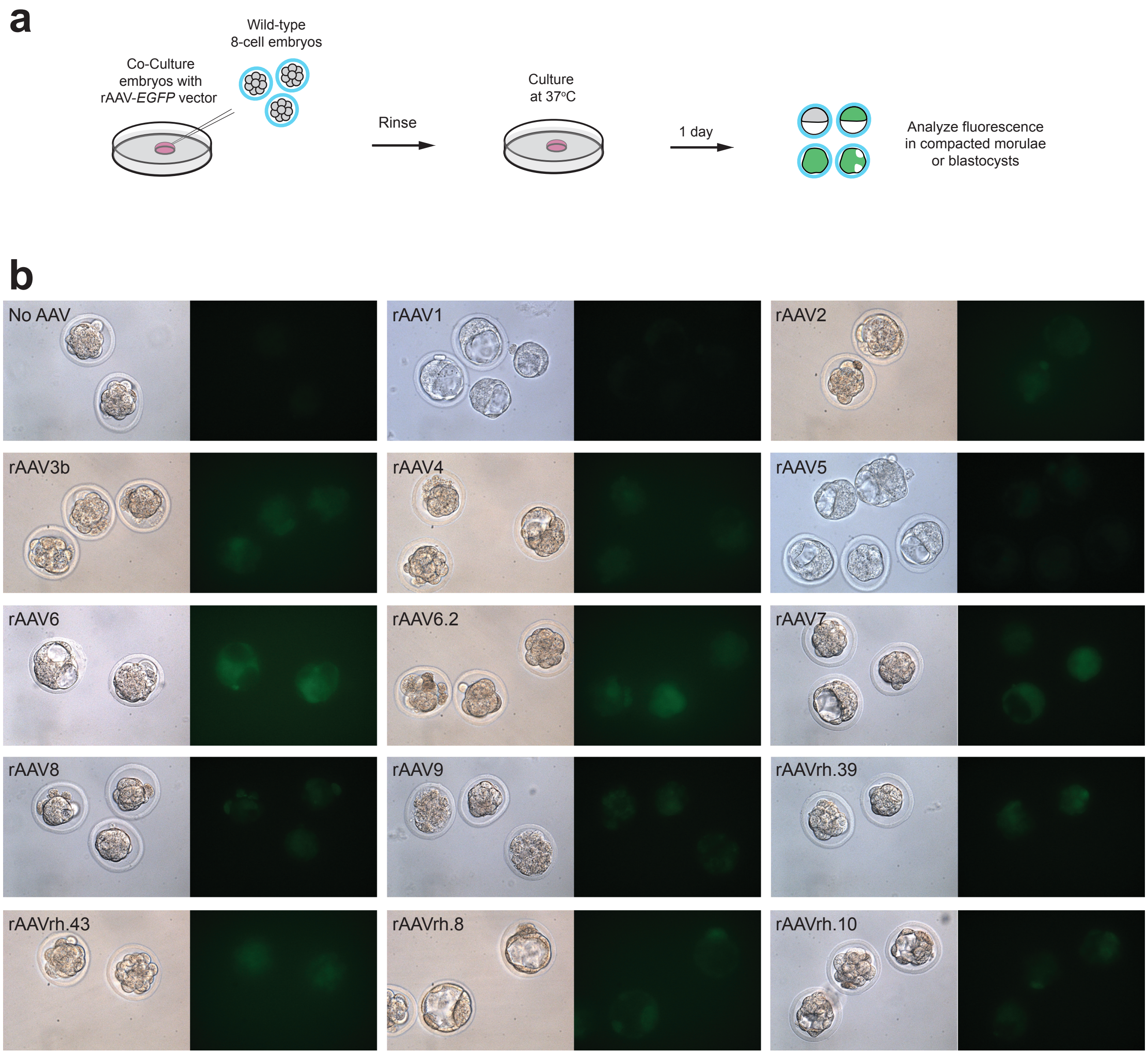
a
Wild-type
8-cell embryos
Co-Culture
embryos with
rAAV-EGFP vector
Rinse
Culture
at 37°C
1 day
Analyze fluorescence
in compacted morulae
or blastocysts
b
No AAV
rAAV1
rAAV2
rAAV3b
rAAV4
rAAV5
rAAV6
rAAV6.2
rAAV7
rAAV8
rAAV9
rAAVrh.39
rAAVrh.43
rAAVrh.8
rAAVrh.10

Supplementary Figure 1 Multiple rAAV serotypes can transduce intact pre-implantation embryos. (**a**) Strategy to transduce 8-cell morulae with rAAVs. (**b**) Analysis of compacted morulae or blastocysts transduced with individual rAAV serotypes. All rAAV serotypes show evidence of transduction as revealed by *EGFP* expression.

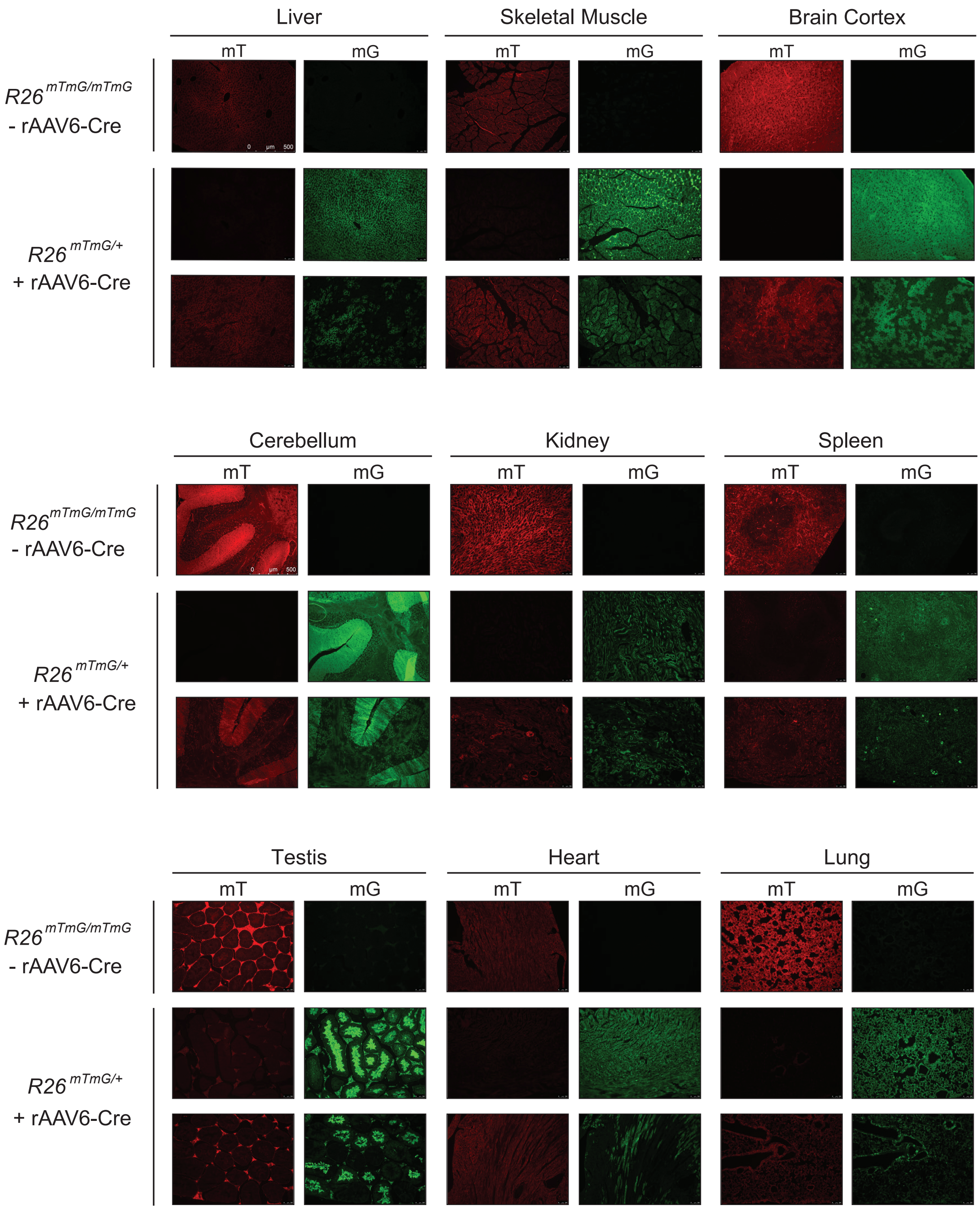
Liver
Skeletal Muscle
Brain Cortex
Cerebellum
Kidney
Spleen
Testis
Heart
Lung
mT
mG
$R26^{mTmG/mTmG}$
- rAAV6-Cre
$R26^{mTmG/+}$
+ rAAV6-Cre
0 μm 500

Supplementary Figure 2 Histological analysis of tissues of adult *R26*^{mTmG} reporter mice transduced with rAAV6-*Cre* at zygote stage. Representative fluorescence images of tissue cryosections from a non-transduced control *R26*$^{mTmG/mTmG}$ mouse (top rows), *R26*$^{mTmG/+}$ mice transduced with *rAAV6-Cre* with complete Cre recombination (middle rows) and *R26*$^{mTmG/+}$ mice transduced with *rAAV6-Cre* with partial Cre recombination (bottom rows). Derivatives of all three germ layers are shown. Slight red fluorescence observed in the middle row of testis section is the result of auto-fluorescence[1].

a

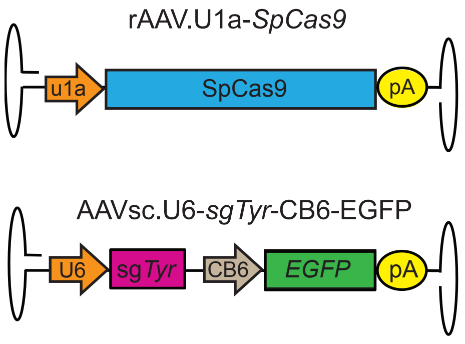

b

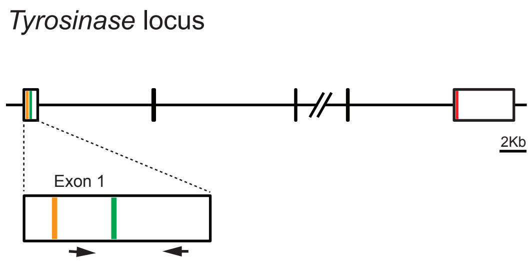

c

5'TTGTTGGCAAAAGAATGCTGCCCACCATGGATGGGTGATGGGAGTCCCTGCGGCCAGC
TTTCAGGCAGAGGTTCCTGCCAGGATATCCTTCTGTCCAGTGCACCATCTGGACCTCAGT
TCCCCTTCAAAGGGGTGGATGACCGTGAGTCCTGGCCCTCTGTGTTTTATAATAGGACCT
GCCAGTGCTCAGGC**AACTTCATGGGTTTCAACTG**CGGAAACTGTAAGTTTGGATTTGGGG
GCCCAAATTGTACAGAGAAGCGAGTCTTGATTAGAAGAAACATTTTTGATTTGAGTGTCT
CCGAAAAGAATAAGTTCTTTTCTTACCTCACTTTAGCAAAACATACTATCAGCTCAGTCT
ATGTCATCCCCACAGGCACCTATGGCCAAATGAACAATGGGTCAACACCCATGTTTAATG
ATATCAACATCTACGACCTCTTTGTATGGATGCATTACTATGTGTCAAGGGACACACTGC
TTGGGGGCTCTGAAATATGGAGGGACATTGATTTTGCCCATGAAGC-3'

d

ID	guide RNA and PAM	orientation	T7EI bands (bp)
1	TCAGTTCCCCTTCAAAGGGGTGG	sense	394, 130
2	GGTCCTATTATAAAACACAGAGG	antisense	364, 160
3	GGTCATCCACCCCTTTGAAGGGG	antisense	399, 125
**4**	**AACTTCATGGGTTTCAACTGCGG**	**sense**	**315, 209**
5	GGGTGGATGACCGTGAGTCCTGG	sense	377, 147

e

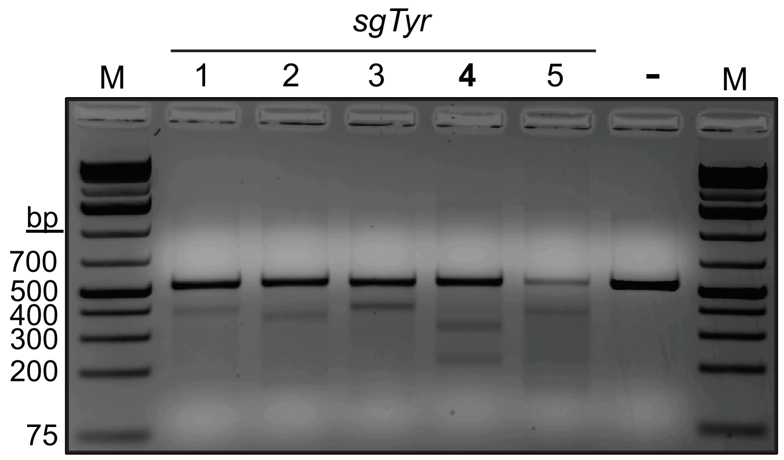

Supplementary Figure 3 Strategy for *Tyr* gene editing using CRISPR-*Cas9* and validation in cell culture. (**a**) Schematic diagrams showing rAAV.U1a-Sp*Cas9* and AAVsc.U6-sg*Tyr*.CB6-*EGFP* vector constructs. Each construct is flanked by inverted terminal repeats (ITR, T-shaped structures). pA: polyadenylation signal from rabbit beta-globin gene. (**b**) Schematic diagram showing the genomic region of mouse *Tyr* gene targeted by CRISPR-Cas9. Orange bar, start codon; Red bar, stop codon; Green bar, region containing targets of sgRNAs; Arrows, T7EI PCR primer binding sites. (**c**) Sequence of mouse *Tyr* region flanked by PCR primers shown in (b) (524 bp). The binding site of sgRNA4 is highlighted in bold; PCR primer sites are underlined and PAM location is shown in red. The location of previously reported $Tyr^{c\text{-}2j}$ and Tyr^{c} mutations is marked in blue and green, respectively. (**d**) Sequence of five different sgRNAs designed to target exon1 of *Tyr* (green region in b). The PAM sequence of each sgRNA is underlined. The orientation and predicted sizes of cleavage bands following T7EI assay are shown. (**e**) Results of T7EI assay to validate the five different sgRNAs (shown in d) in GreenGo cells. sgRNA4 (bold in d) was the most efficient and was chosen for embryo experiments.

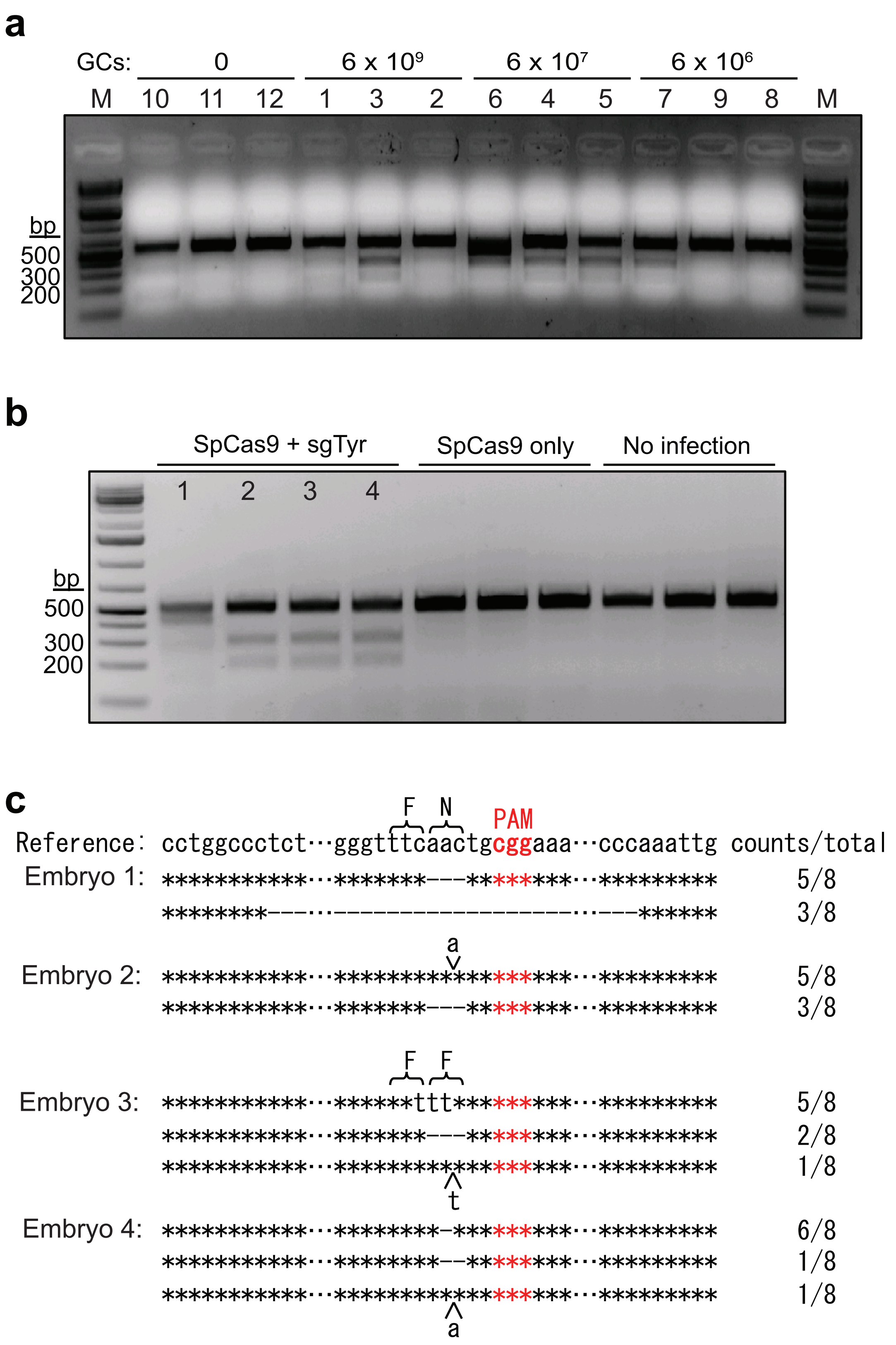

a
GCs: 0 6 x 10⁹ 6 x 10⁷ 6 x 10⁶
M 10 11 12 1 3 2 6 4 5 7 9 8 M
bp
500
300
200
b
SpCas9 + sgTyr
SpCas9 only
No infection
1 2 3 4
bp
500
300
200
c
F N
PAM
Reference: cctggccctct···gggtttcaactgcggaaa···cccaaattg counts/total
Embryo 1: 5/8
3/8
a
Embryo 2: 5/8
3/8
F F
Embryo 3: 5/8
2/8
1/8
t
Embryo 4: 6/8
1/8
1/8
a

Supplementary Figure 4 Analysis of *Tyr* gene editing in mouse embryos using sgRNA4. (**a**) Results of T7EI assay to detect indels in E3.5 embryos that were transduced with different doses of rAAV6-*Cas9* and rAAV6-sg*Tyr* (1:1 ratio) at zygote stage. The dose is the total genome copies (GCs) of the two rAAV vectors contained in a drop of 15 μl of KSOM. Non-transduced embryos served as negative controls. Each lane represents the result of an individual embryo shown in **Figure 1b**. (**b**) Results of T7EI assay to detect indels in E16.5 embryos transduced with 6.0E+9 GCs of rAAV6-*Cas9* and rAAV6-sg*Tyr* (SpCas9+sg*Tyr*) at zygote stage. Embryos that were infected with rAAV6-*Cas9* only (SpCas9 only) or not infected (no infection) served as negative controls. Each lane represents the result of an individual embryo. (**c**) TOPO cloning and Sanger sequencing results of PCR products from the four embryos in the SpCas9+sgTyr group shown in (b). The PAM sequence is colored in red. Coding sequences for amino acid residues phenylalanine (F) and asparagine (N) are labeled. The asterisks represent the same sequence as reference; dashes, deletion mutations and arrowheads, insertion mutations. Eight TOPO clones were picked and sequenced for each embryo. Counts of each unique read are shown far right.

a

Sample ID	Barcode & primer (5'→3')	Barcode alone (5'→ 3')
Tyr_Fwd_10	GCGCTCTGTGTGCAGCTTGTTGGCAAAAGAATGCTG	GCGCTCTGTGTGCAGC
Tyr_Fwd_11	TCATGAGTCGACACTATTGTTGGCAAAAGAATGCTG	TCATGAGTCGACACTA
Tyr_Fwd_12	TATCTATCGTATACGCTTGTTGGCAAAAGAATGCTG	TATCTATCGTATACGC
Tyr_Fwd_1	ATCACACTGCATCTGATTGTTGGCAAAAGAATGCTG	ATCACACTGCATCTGA
Tyr_Fwd_3	ACGTACGCTCGTCATATTGTTGGCAAAAGAATGCTG	ACGTACGCTCGTCATA
Tyr_Fwd_2	TGTGAGTCAGTACGCGTTGTTGGCAAAAGAATGCTG	TGTGAGTCAGTACGCG
Tyr_Fwd_6	AGAGACACGATACTCATTGTTGGCAAAAGAATGCTG	AGAGACACGATACTCA
Tyr_Fwd_4	CTGCTAGAGTCTACAGTTGTTGGCAAAAGAATGCTG	CTGCTAGAGTCTACAG
Tyr_Fwd_5	AGCACTCGCGTCAGTGTTGTTGGCAAAAGAATGCTG	AGCACTCGCGTCAGTG
Tyr_Fwd_7	TCATGCACGTCTCGCTTTGTTGGCAAAAGAATGCTG	TCATGCACGTCTCGCT
Tyr_Fwd_9	AGAGCATCTCTGTACTTTGTTGGCAAAAGAATGCTG	AGAGCATCTCTGTACT
Tyr_Fwd_8	CGCATCGACTACGCTATTGTTGGCAAAAGAATGCTG	CGCATCGACTACGCTA
Tyr_Rev_10	AGAGTACTACATATGAGCTTCATGGGCAAAATCAAT	AGAGTACTACATATGA
Tyr_Rev_11	CGTGTGCATAGATCGCGCTTCATGGGCAAAATCAAT	CGTGTGCATAGATCGC
Tyr_Rev_12	ATGTATCTCGACTGCAGCTTCATGGGCAAAATCAAT	ATGTATCTCGACTGCA
Tyr_Rev_1	GACTCGACGCAGAGTCGCTTCATGGGCAAAATCAAT	GACTCGACGCAGAGTC
Tyr_Rev_3	CGATGACGTCGCTGTAGCTTCATGGGCAAAATCAAT	CGATGACGTCGCTGTA
Tyr_Rev_2	CACACGTAGTCTGCGCGCTTCATGGGCAAAATCAAT	CACACGTAGTCTGCGC
Tyr_Rev_6	GCTGTATCGCAGAGACGCTTCATGGGCAAAATCAAT	GCTGTATCGCAGAGAC
Tyr_Rev_4	CGAGCTATCTCATACTGCTTCATGGGCAAAATCAAT	CGAGCTATCTCATACT
Tyr_Rev_5	CATGAGTACTCGTCGCGCTTCATGGGCAAAATCAAT	CATGAGTACTCGTCGC
Tyr_Rev_7	CAGCGACTGTGATACTGCTTCATGGGCAAAATCAAT	CAGCGACTGTGATACT
Tyr_Rev_9	TGTCGCATCATATGATGCTTCATGGGCAAAATCAAT	TGTCGCATCATATGAT
Tyr_Rev_8	GCTGTGATCTACGTCTGCTTCATGGGCAAAATCAAT	GCTGTGATCTACGTCT

b

Detected unique reads	Indel-type
GCTCAGGCAACTTCATGGGTTTCAA CTGCGGAAACTGTAAGTTTGGATTT	Reference
GCTCAGGCAACTTCATGGGTTTCAAA CTGCGGAAACTGTAAGTTTGGATTT	insertion
GCTCAGGCAACTTCATGGGTTTCAAAACTGCGGAAACTGTAAGTTTGGATTT	insertion
GCTCAGGCAACTTCATGGGTTTCAAATCTGCGGAAACTGTAAGTTTGGATTT	insertion
GCTCAGGCAACTTCATGGGTTTCAAT CTGCGGAAACTGTAAGTTTGGATTT	insertion
GCTCAGGCAACTTCATGGGTTT--- CTGCGGAAACTGTAAGTTTGGATTT	deletion
GCTCAGGCAACTTCATGGGTTTC-- -TGCGGAAACTGTAAGTTTGGATTT	deletion
GCTCAGGCAACTTCATGGGTTTC-- CTGCGGAAACTGTAAGTTTGGATTT	deletion
GCTCAGGCAACTTCATGGGTTTCA- -TGCGGAAACTGTAAGTTTGGATTT	deletion
GCTCAGGCAACTTCATGGGTTTCA- CTGCGGAAACTGTAAGTTTGGATTT	deletion
GCTCAGGCAACTTCATGGGTTTCAA -TGCGGAAACTGTAAGTTTGGATTT	deletion
GCTCAGGCAACTTCATGGGTTTCAA CT--GGAAACTGTAAGTTTGGATTT	deletion
GCTCAGGCAACTTCATGGGTTTCAT CTGCGGAAACTGTAAGTTTGGATTT	alteration
--G	large deletion
-----------------------------GCGGAAACTGTAAGTTTGGATTT	large deletion
GCTCAGGCAACTTCATGGGTTT--A CTGCGGAAACTGTAAGTTTGGATTT	compound
GCTCAGGCAACTTCATGGGTTTC-T CTGCGGAAACTGTAAGTTTGGATTT	compound
GCTCAGGCAACTTCATGGGTTTCA- --TTGGAAACTGTAAGTTTGGATTT	compound
GCTCAGGCAACTTCATGGGTTTCAA ----TGAAACTGTAAGTTTGGATTT	compound
GCTCAGGCAACTTCATGGGTTTCAA ---TGGAAACTGTAAGTTTGGATTT	compound
GCTCAGGCAACTTCATGGGTTTCAT ----GGAAACTGTAAGTTTGGATTT	compound
GCTCAGGCAACTTCATGGGTTTCAT CT--GGAAACTGTAAGTTTGGATTT	compound

Supplementary Figure 5 SMRT sequencing of CRISPR-Cas9-induced *Tyr* indel events. (**a**) Table of asymmetrically indexed primer sets for *Tyr* gene PCR. Sequences in red indicate nucleotides that match the *Tyr* locus. (**b**) Summary table of unique indel/editing events detected by SMRT sequencing of all sample libraries derived from E3.5 embryos shown in **Figure 1c**. The PAM sequence is indicated in green, hyphens (-) mark deletion events, and red bases indicate the insertion or alteration of base(s).

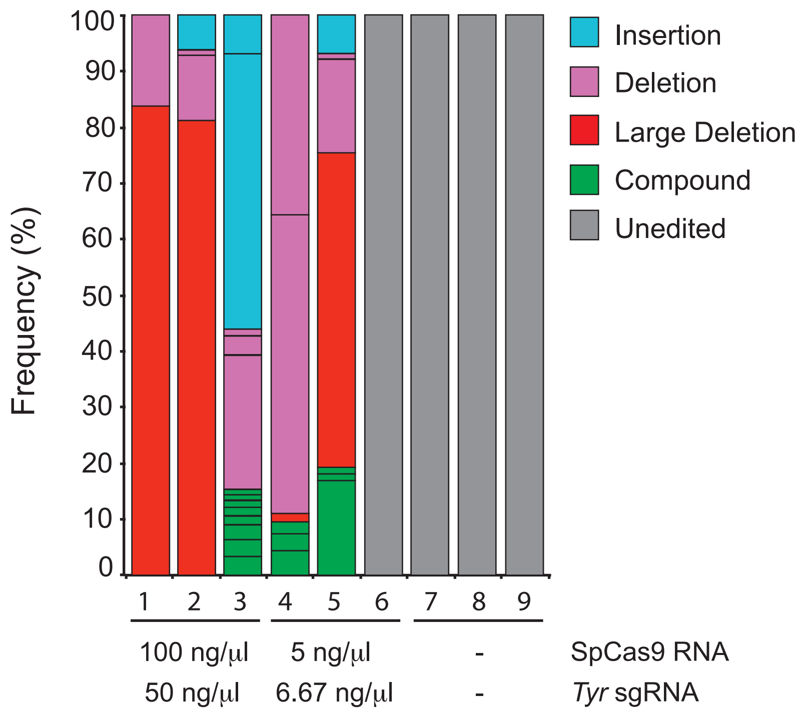

Frequency (%)
100
90
80
70
60
50
40
30
20
10
0
1
2
3
4
5
6
7
8
9
Insertion
Deletion
Large Deletion
Compound
Unedited
100 ng/μl
5 ng/μl
-
SpCas9 RNA
50 ng/μl
6.67 ng/μl
-
Tyr sgRNA

Supplementary Figure 6. Gene editing at the *Tyrosinase* locus using pronuclear injection technique. Stacked histogram showing the percentage distribution of indel-type frequencies from zygotes injected with two different mixtures of *Tyr* sgRNA and Cas9 mRNA and analyzed at compacted morula or blastocyst stages. Each bar indicates the frequency of reads detected by SMRT sequencing for unique indels at the target locus encountered per embryo. Three groups of embryos are depicted: embryos with high dose of *Tyr* sgRNA and Cas9 RNA (1-3), embryos with low dose (4-6) and non-treated embryos. Alterations indicate base replacements; large Deletions are defined as removal of >20 bases and compound mutations are combinations of insertions, deletions, and/or alterations.

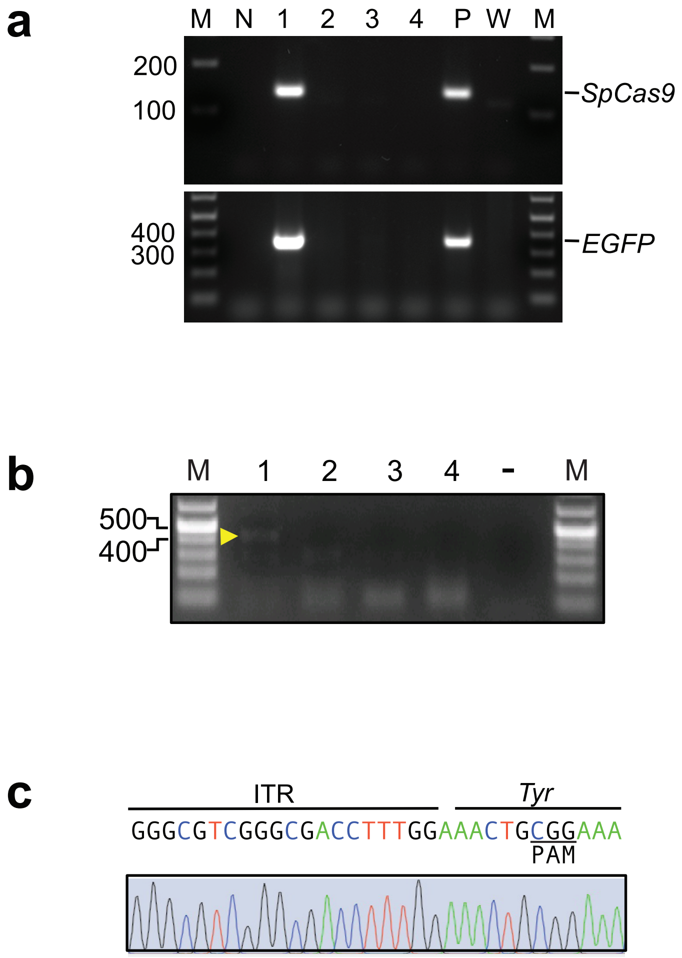
a
M N 1 2 3 4 P W M
200
100
SpCas9
400
300
EGFP
b
M 1 2 3 4 - M
500
400
c
ITR
Tyr
GGGCGTCGGGCGACCTTTGGAAACTGCGGAAA
PAM

Supplementary Figure 7 Analysis of rAAV6 genome integration into the *Tyr* gene following SpCas9-mediated cleavage. (**a**) Results of PCR to amplify *SpCas9* gene (top row) and *EGFP* gene (bottom row) using DNA of tail snips from four albino mice generated by rAAV6.u1a-SpCas9/rAAV6.U6-sgRNA.CB6-EGFP transduction of C57BL/6NJ zygotes. Mouse 1 shows bands from each PCR, suggesting that this mouse contains the genome of both *rAAV6.u1a-SpCas9* and *rAAV6.U6-sgRNA.CB6-EGFP*. M, 1kb plus DNA ladder. N, no template. P, positive control. W, wildtype sample. (**b**) PCR to detect rAAV genome integration into the *Tyr* gene at the predicted SpCas9 cleavage site in the same albino pups analyzed in (a). The yellow arrowhead indicates a positive band from mouse 1. (**c**) TOPO cloning of the highlighted band in (b) and Sanger sequencing results with chromatogram. The depicted sequence demonstrates fusion of the rAAV-ITR and the *Tyr* gene. The PAM sequence for *sgTyr* is also labeled (underlined).

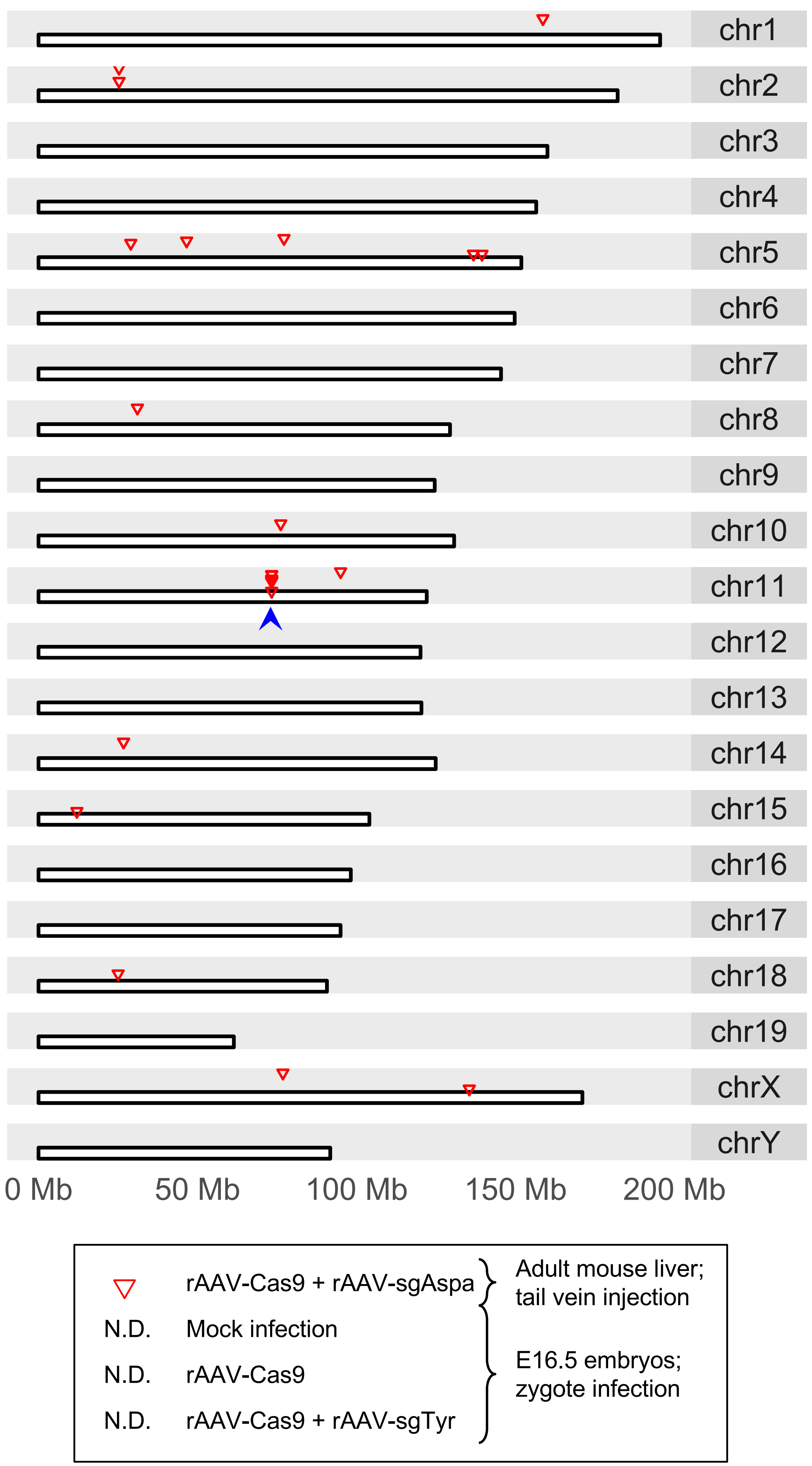
chr1
chr2
chr3
chr4
chr5
chr6
chr7
chr8
chr9
chr10
chr11
chr12
chr13
chr14
chr15
chr16
chr17
chr18
chr19
chrX
chrY
0 Mb
50 Mb
100 Mb
150 Mb
200 Mb
rAAV-Cas9 + rAAV-sgAspa
N.D.
Mock infection
N.D.
rAAV-Cas9
N.D.
rAAV-Cas9 + rAAV-sgTyr
Adult mouse liver; tail vein injection
E16.5 embryos; zygote infection

Supplementary Figure 8 Treatment of embryos with rAAV-Cas9/sg*Tyr* does not lead to detectable levels of rAAV integration. Karyogram display showing the detection of integration events across the mouse genome in a control sample targeted at the *Aspartoacylase* (*Aspa*) gene. Tail vein injection of rAAV9-*Cas9*/*sgAspa* into an adult mouse results in the detection of multiple integration events in the liver (red triangles, n=1), serving as positive control for the rAAV integration analysis method. Location of the *Aspa* gene on chromosome 11 is indicated by the blue arrowhead. To detect possible rAAV integration following zygote infection, DNA from whole E16.5 embryos was analyzed. Mock infection, infection with Cas9 vector alone, and coinfection with Cas9 and sgTyr vectors did not result in detectable levels of integration when analyzed in E16.5 embryos (N.D. = not detected). n=3.

a

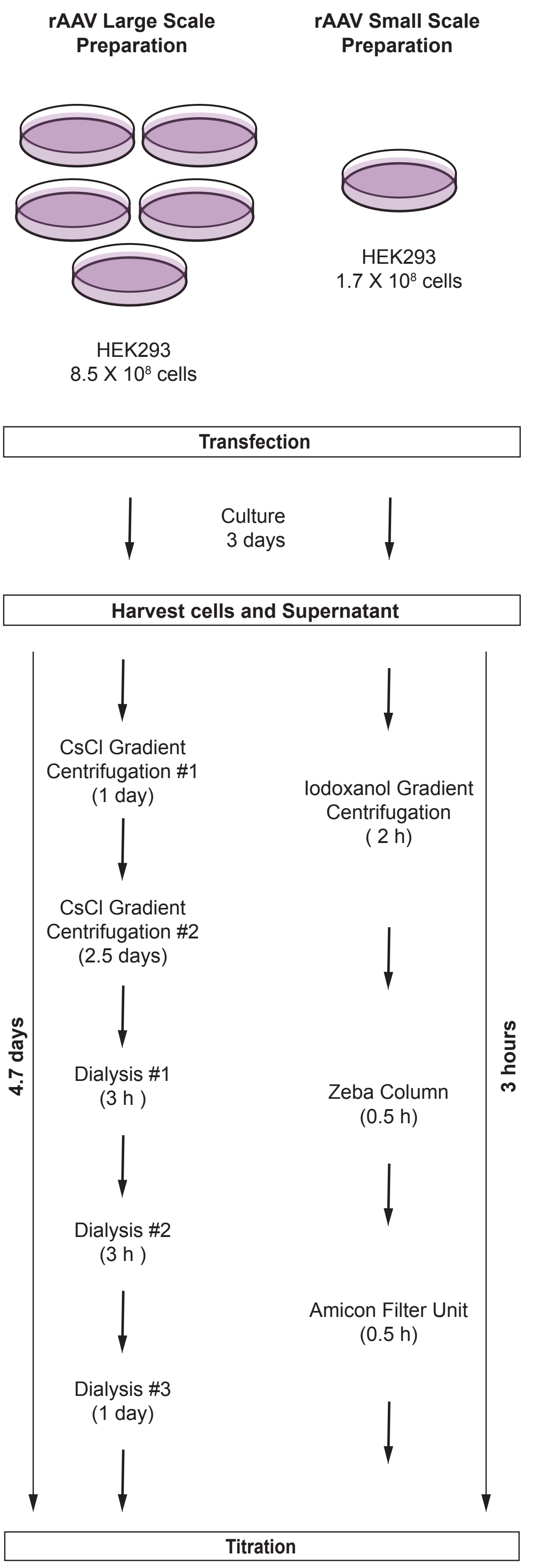

b

Large Scale (Dose)	Small Scale (Dose)	Embryo ID	Edited/ Total Clones	Gene Editing (Frequency)
rAAV6-*Cas9* (1 x 10^9 GCs)	rAAV6-*sgTyr* (1 x 10^9 GCs)	1	6/7	56/58 (97%)
		2	6/7	
		3	8/8	
		4	8/8	
		5	6/6	
		6	8/8	
		7	8/8	
		8	6/6	

c

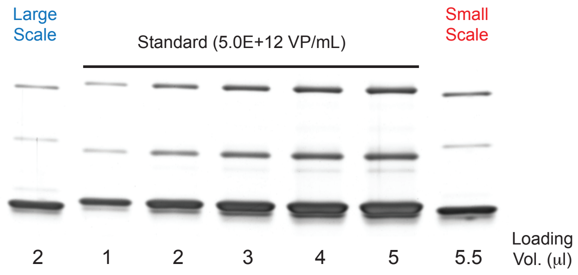

d

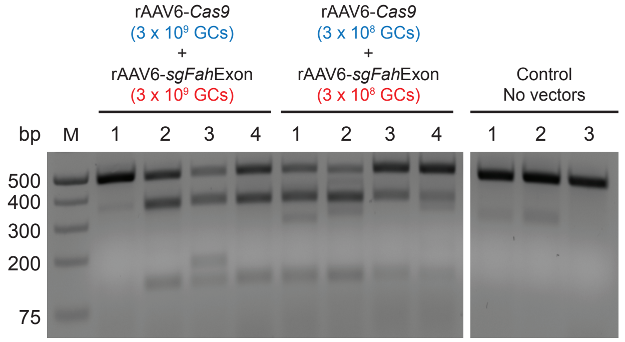

e

Large Scale (Dose)	Small Scale (Dose)	Embryo ID	Edited/ Total Clones	Gene Editing (Frequency)
rAAV6-*Cas9* (3 x 10^9 GCs)	rAAV6-*sgFah*Exon (3 x 10^9 GCs)	1	7/7	29/29 (100%)
		2	7/7	
		3	7/7	
		4	8/8	
rAAV6-*Cas9* (3 x 10^8 GCs)	rAAV6-*sgFah*Exon (3 x 10^8 GCs)	1	8/8	25/27 (93%)
		2	8/8	
		3	5/5	
		4	4/6	

Supplementary Figure 9 Genome editing using small scale preparation of rAAV vector. (a) Schematic representation of the large and small scale methods used to purify rAAV vectors. **(b)** TOPO sequencing analysis of *Tyr* gene editing in E3.5 embryos that were infected with the large-scale rAAV6-Cas9 and small-scale rAAV6-sg*Tyr* vector preparations. (**c**) Gel image of silver staining of rAAV6-sg*Fah*Exon vectors prepared by the large-scale protocol (far left) and small-scale protocol (far right), together with a standard rAAV2 vector (5.0E+12 viral particles per milliliter, VP/mL) loaded at escalating amounts. Only three viral proteins, VP1, VP2, and VP3, are seen from top to bottom, indicating the purity of all rAAV vectors. (**d**) T7EI nuclease analysis of *Fah* gene editing in E3.5 embryos that were infected with the large-scale rAAV6-Cas9 and small-scale rAAV6-sg*Fah*Exon at two doses. Embryos that were not infected with rAAV serve as negative control. (**e**) Summary table showing *Fah* gene editing efficiency in eight embryo samples as determined by TOPO sequencing. Five to eight TOPO clones were sequenced for each embryo. Gene editing efficiency is calculated as the ratio of edited clones over total clones sequenced within each rAAV dose group.

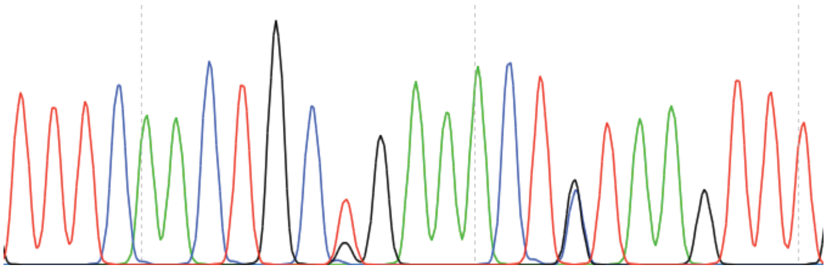

Tyr^{SNT} TTTCAACTGCTGAAACTGTAAGTTT

Tyr^{c} TTTCAACTGCGGAAACTCTAAGTTT

Tyr^{+} TTTCAACTGCGGAAACTGTAAGTTT

Supplementary Figure 10 Representative chromatogram showing the presence of the *Tyr*^{SNT} allele in F1 pups. The SNT-positive founder mouse shown in Fig. 3d was bred with a CD-1 female (*Tyr*$^{c/c}$). DNA was extracted from tail snips of F1 pups and subjected to PCR using primers located outside of the region of homology (Fig. 3b). The resulting PCR amplicons from individual pups were sequenced. The overlapping peaks demonstrate the presence of the G->T transversion (red T) inherited from the F0 founder, and the “C” mutation (blue C) inherited from the CD-1 female. The wild-*type* Tyrosinase sequence (*Tyr*$^{+}$) is provided for reference.
